# Genome-Wide Identification, Evolution, and Comparative Analysis of B-Box Genes in *Brassica rapa*, *B. oleracea*, and *B. napus* and Their Expression Profiling in *B. rapa* in Response to Multiple Hormones and Abiotic Stresses

**DOI:** 10.3390/ijms221910367

**Published:** 2021-09-26

**Authors:** Sonam Singh, Sushil Satish Chhapekar, Yinbo Ma, Jana Jeevan Rameneni, Sang Heon Oh, Jusang Kim, Yong Pyo Lim, Su Ryun Choi

**Affiliations:** 1 Department of Horticulture, College of Agriculture and Life Science, Chungnam National University, Daejeon 34134, Korea; sonamsingh688@gmail.com (S.S.); sushilchhapekar@gmail.com (S.S.C.); mayinbo@126.com (Y.M.); saijeevan7@gmail.com (J.J.R.); rederaser@naver.com (S.H.O.); 2 Breeding Research Institute, Dayi International Seed Co., Ltd., 16-35 Ssiat-gil, Baeksan-myeon, Gimje 54324, Jeollabuk-do, Korea; juzu1227@dayiseed.co.kr

**Keywords:** B-box, *BBX*, *Brassica rapa*, Brassicaceae, evolution, gene duplication, synteny, abiotic stress, hormonal stress

## Abstract

The B-box zinc-finger transcription factors are important for plant growth, development, and various physiological processes such as photomorphogenesis, light signaling, and flowering, as well as for several biotic and abiotic stress responses. However, there is relatively little information available regarding *Brassica* B-box genes and their expression. In this study, we identified 51, 52, and 101 non-redundant genes encoding B-box proteins in *Brassica rapa* (*BrBBX* genes), *B. oleracea* (*BoBBX* genes), and *B. napus* (*BnBBX* genes), respectively. A whole-genome identification, characterization, and evolutionary analysis (synteny and orthology) of the B-box gene families in the diploid species *B. rapa* (A genome) and *B. oleracea* (C genome) and in the allotetraploid species *B. napus* (AC genome) revealed segmental duplications were the major contributors to the expansion of the *Brassica* *BBX* gene families. The *Brassica* *BBX* genes were classified into five subgroups according to phylogenetic relationships, gene structures, and conserved domains. Light-responsive cis-regulatory elements were detected in many of the *BBX* gene promoters. Additionally, *BrBBX* expression profiles in different tissues and in response to various abiotic stresses (heat, cold, salt, and drought) or hormones (abscisic acid, methyl jasmonate, and gibberellic acid) were analyzed by qRT-PCR. The data indicated that many B-box genes (e.g., *BrBBX13*, *BrBBX15*, and *BrBBX17*) may contribute to plant development and growth as well as abiotic stress tolerance. Overall, the identified BBX genes may be useful as functional genetic markers for multiple stress responses and plant developmental processes.

## 1. Introduction

The B-box (*BBX*) genes have recently been revealed to encode multifarious zinc-finger transcription factors with diverse functions in several plant species [1,2]. The *BBX* transcription factor family members are major contributors to several activities, including the photoperiod-related regulation of flowering, plant developmental processes, seedling photomorphogenesis, seed germination, and abiotic and biotic stress responses [3]. Plant *BBX* transcription factors have one or two N-terminal BBX domains with or without a C-terminal CCT domain (COL-like/CO or TOC1 motif), unlike the corresponding proteins in animals, which include a RING finger and a coiled-coil domain along with the BBX domain.

**Figure 1:**
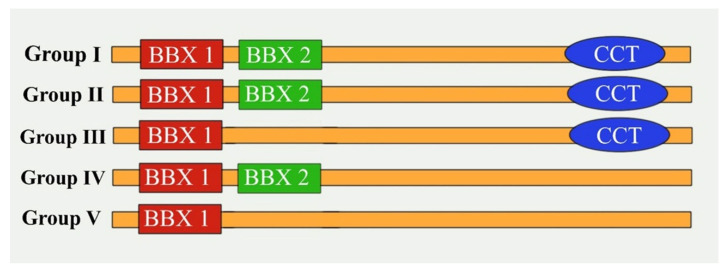
Scheme illustrating major features of BBX proteins in each structure group (Modified from Talar et al. [11]).

In plants, based on the distance between the zinc-binding residues and their consensus sequence, the BBX domains can be divided into the B-box1 (B1) and B-box2 (B2) classes [4]. The CCT domain, which is highly conserved and composed of 42 or 43 amino acids, is important for nuclear protein transport and the regulation of transcription [5,6]. The BBX domain is predicted to include conserved histidine (H), cysteine (C), and aspartic acid (D) residues that modulate protein–protein interactions [4,7,8]. Khanna et al. identified 32 *BBX* genes in *Arabidopsis thaliana* and named them *AtBBX1*–*32* [4]. These 32 BBX genes were further classified into Groups I–V depending on the number of BBX and CCT domains [3,4,9] (Figure 1). In *A. thaliana*, *CONSTANS* (*CO*)/*AtBBX1*, which was the first member of the BBX family to be studied, is the main coordinator of flowering time because it induces *FLOWERING LOCUS T* (*FT*) expression [9,10].

In transgenic *A. thaliana* plants, the overexpression of the *CO* gene results in abnormal flowering under short-day and long-day conditions, whereas a mutation of this gene delays flowering under long-day conditions [12,13,14]. Approximately 10 *BBX* genes have been identified as crucial regulators of early photomorphogenesis, including *BBX4*, *BBX7*, *BBX21*, and *BBX32*, which control the flowering time [15,16,17]. Previous studies on *A. thaliana* revealed that *BBX19*, *BBX20*, *BBX24*, *BBX25*, *BBX28*, and *BBX32* negatively regulate photomorphogenesis [18,19,20], whereas *BBX4*, *BBX21*, *BBX22*, and *BBX23* have the opposite effect [21,22,23]. Various reports confirmed that BBX proteins associate with *ELONGATED HYPOCOTYL 5* (*HY5*) at the protein or gene level and modulate plant photomorphogenesis. For example, in response to irradiation, *BBX21* binds to *BBX22* and *HY5* as well as its own promoter and activates transcription, whereas *BBX11* binds to the *HY5* promoter to modulate expression [21,22,24,25]. Intriguingly, in *A. thaliana* exposed to red light, *BBX4* accumulates and interacts with *phyB*, thereby enhancing photomorphogenesis [26]. A similar mechanism was also detected in other plant species. Specifically, in apple, *MdHY5* promoter activity is inhibited by *MdBBX37*. Additionally, *MdHY5* expression is modulated by *MdBBX25*/*MdCOL4* and *MdBBX22*, which binds directly to the promoter and decreases or increases transcription [27,28]. In rice, *OsHY5L1* expression is induced by *OsBBX14* to positively affect photomorphogenesis [29]. In tomato, *SlBBX20* interacts with the *PSY1* promoter, which enhances carotenoid biosynthesis [30].

BBX proteins might also have an important role in abiotic stress responses and hormone signaling networks. In tomato, rice, apple, and grapevine, some stress- and hormone signaling-related cis-elements, such as the LTR, MBS, HSE, ABA-responsive element (ABRE), ethylene-responsive element (ERE), and gibberellin-responsive element (GARE), have been detected in the promoter region of most *BBX* genes, implying these genes are involved in hormone signaling pathways and abiotic stress responses [8,31,32,33] However, their actual functions related to abiotic stress responses and hormone signaling pathways remain unknown. The *A. thaliana AtBBX24* gene encodes a salt tolerance protein (STO), which can increase the salt tolerance of transgenic yeast cells. Furthermore, the overexpression of *AtBBX24* in *A. thaliana* leads to significantly increased salt tolerance and root growth [34,35]. In chrysanthemum, *CmBBX24* delays flowering and increases cold and drought tolerance [9]. In *A. thaliana*, STO interacts with CLONE EIGHTY ONE/RADICAL-INDUCED CELL DEATH1 (CEO/RCD1), which negatively regulates the expression of a broad range of stress-related genes [36,37]. Another *A. thaliana* BBX family member, *AtBBX18*, negatively regulates both heat tolerance and photomorphogenesis, whereas in grapevine, *VvBBX32* overexpression results in cold stress tolerance [38,39]. Moreover, *AtBBX18* downregulates the expression of other heat-responsive genes, such as *Hsp70*, *DGD1*, *APX2*, and *Hsp101*, which adversely affects seedling survival and germination under heat stress conditions [39]. Other studies demonstrated that *AtBBX18* functions as a positive regulator in the gibberellin (GA) signaling network, but *AtBBX20* is a negative regulator in the brassinosteroid signaling pathway [18,40].

The genus *Brassica* comprises approximately 100 species, including important vegetable and oilseed crops cultivated worldwide. Chinese cabbage (*Brassica rapa* L.), broccoli (*Brassica oleracea* L.), and rapeseed (*Brassica napus* L.) are mostly cultivated as vegetables for human consumption and for producing oil, condiments, and fodder because of their nutrient contents, including vitamins, proteins, and minerals (e.g., zinc, iron, sodium, and potassium) [41,42,43].

To date, *BBX* genes have been identified in numerous plant species, including rice [8], soybean [44], tomato [31], pear [45,46], potato [47], apple [32], Rosaceae [48], Poaceae [49], and grapevine [33]. Although there are several studies on *BBX* genes in many plants, they have not been comprehensively characterized in *Brassica* species and their evolution and functions related to abiotic and hormone stress responses have yet to be studied. Therefore, we identified 51 *BBX* genes in *B. rapa*, 52 *BBX* genes in *B. oleracea*, and 101 *BBX* genes in *B. napus* and subsequently analyzed their evolutionary relationships, structures, expression patterns, cis-elements, and chromosomal locations as well as their effects on various abiotic and hormone stress responses. The results of this study will provide new insights regarding the evolutionary importance of the functional divergence of these genes and will lay the foundation for future investigations on the BBX proteins in Brassicaceae.

## 2. Results

### 2.1. Identification of BBX Genes in Brassica Species and Physicochemical Analysis and Subcellular Localization of the Encoded Proteins

The Hidden Markov Model as well as the *A. thaliana BBX* gene sequences obtained from the TAIR database were used to identify the *BBX* gene family members in *B. rapa*, *B. oleracea*, and *B. napus*. The BBX domains of the BBX genes in all three *Brassica* species were identified using the NCBI-CDD, SMART, and Pfam databases. A total of 51, 52, and 101 *BBX* genes were identified in *B. rapa*, *B. oleracea*, and *B. napus*, respectively, after the redundant sequences were eliminated (Table 1). The 204 non-redundant *BBX* genes were renamed as *B. rapa BrBBX01*–*51* (Table S1), *B. oleracea BoBBX01*–*52* (Table S2), and *B. napus BnBBX01*–*101* (Table S3) according to the relative linear order on each chromosome and widely used nomenclature. Furthermore, *BnBBX* referred to the BBX genes in the whole *B. napus* genome, whereas *BnABBX* and *BnCBBX* genes belonged to the A and C genomes, respectively.

The molecular weights of the *Brassica* BBX proteins ranged from 11.253 kDa (BrBBX3) to 61.128 kDa (BrBBX9) in *B. rapa*, from 12.397 kDa (BoBBX3) to 46.391 kDa (BoBBX23) in *B. oleracea*, and from 4.801 kDa (BnABBX28) to 62.649 kDa (BnCBBX69) in *B. napus*. The pI ranged from 11.60 (BnCBBX96) to 3.81 (BnABBX7). On the basis of their pI, most *B. rapa* BBX proteins were acidic; the exceptions were BrBBX3, BrBBX8, BrBBX9, BrBBX13, BrBBX19, BrBBX24, and BrBBX51, which had a pI greater than 7. Regarding *B. oleracea* and *B. napus*, 15 and 30 BBX proteins had a pI exceeding 7, respectively. However, most of the *Brassica* BBX proteins were unstable, with an instability index greater than 40 (Tables S1–S3). The *Brassica* BBX proteins lacked a transmembrane segment, with the exception of BrBBX33 and BnABBX3 (data not shown). The GRAVY index for almost all BBX proteins (<0) reflected the hydrophilic nature of these proteins. Only BnABBX3 had a GRAVY index greater than 0 (0.098). The aliphatic index for all *Brassica* BBX proteins ranged from 46.04 to 85.73. The subcellular localization analysis revealed that the BrBBX proteins were mostly localized in the nucleus (30 BrBBXs), followed by the chloroplast (11 BrBBXs), cytoplasm (9 BrBBXs), and the endoplasmic reticulum (1 BrBBX). In *B. oleracea*, 31, 14, 6, and 1 BBX proteins were localized in the nucleus, chloroplast, cytoplasm, and mitochondria, respectively. In *B. napus*, 62, 22, 12, and 2 BBX proteins were localized in the nucleus, chloroplast, cytoplasm, and mitochondria, respectively, whereas one BBX was localized in the extracellular matrix, vacuole, and endoplasmic reticulum (Tables S1–S3). The most common amino acids in the *Brassica* BBX proteins were serine (S), alanine (A), leucine (L), and glutamic acid (L), whereas threonine (T), cysteine (C), and proline (P) were the least abundant amino acids (Tables S1–S3).

### 2.2. Analyses of the Phylogenetic Relationships, Conserved Domains, Structures, and Motifs of B. rapa, B. oleracea, and B. napus BBX Genes

**Figure 2:**
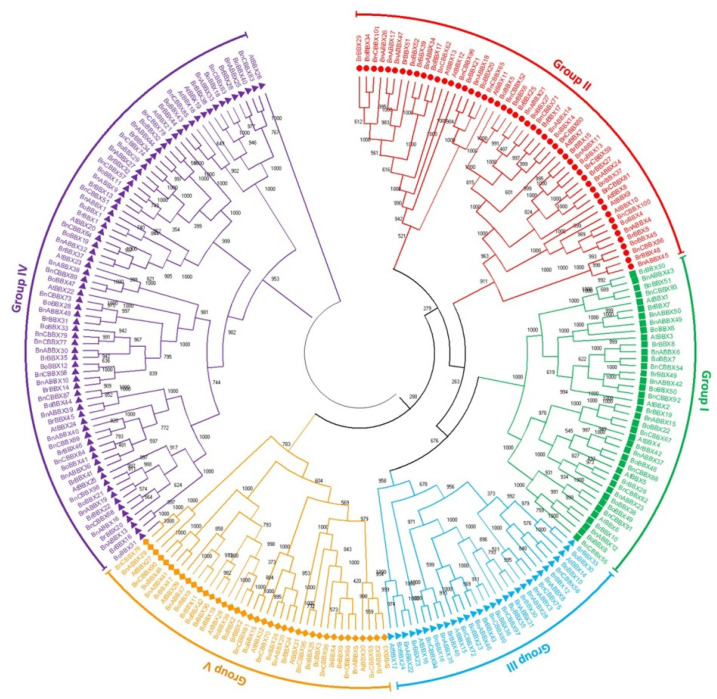
Phylogenetic tree of *B-box* genes from *Brassica rapa*, *B. napus*, *B. oleracea*, and *Arabidopsis thaliana.* The phylogenetic tree was constructed according to the neighbor-joining method. The unrooted tree was generated using MEGA5 on the basis of B-box amino acid sequences from *B. rapa*, *B. napus*, *B. oleracea*, and *A. thaliana.* Roman numerals (I–V) represent different gene clusters. The genes from each species are differentiated by color. The numbers at the nodes represent bootstrap percentage values.

To elucidate the evolutionary relationships and functional divergence among *Brassica* BBX proteins, the sequences of 51 *B. rapa* BBX proteins, 52 *B. oleracea* BBX proteins, 101 *B. napus* BBX proteins, and 32 *A. thaliana* BBX proteins were used to construct an unrooted phylogenetic tree (Figure 2). The BBX proteins were divided into five groups (Groups I–V) according to previous studies [3,4]. The Group I BBXs contained B1 and B2 domains along with the CCT domain; the Group II BBXs had the same domains as the Group I proteins, but the B2 domain differed at the amino acid level; the Group III BBXs included the B1 domain and the CCT domain; the Group IV BBXs had B1 and B2 domains, but lacked the CCT domain; and the Group V BBXs only had a single B1 domain. Groups I and IV had the most BBX members (Figure 2). Some of the BBXs were not categorized as expected. For example, *BrBBX5*, *BrBBX48*, *BoBBX4*, and *BoBBX45* included only the B1 domain and the CCT domain, suggesting they should belong to Group III, but they were in Group II in the phylogenetic tree; likewise, *BrBBX16* and *BnCBBX96* should be in Group V as they included only the B1 domain, but they fall in Group IV and II, respectively (Figure 2).

**Figure 3:**
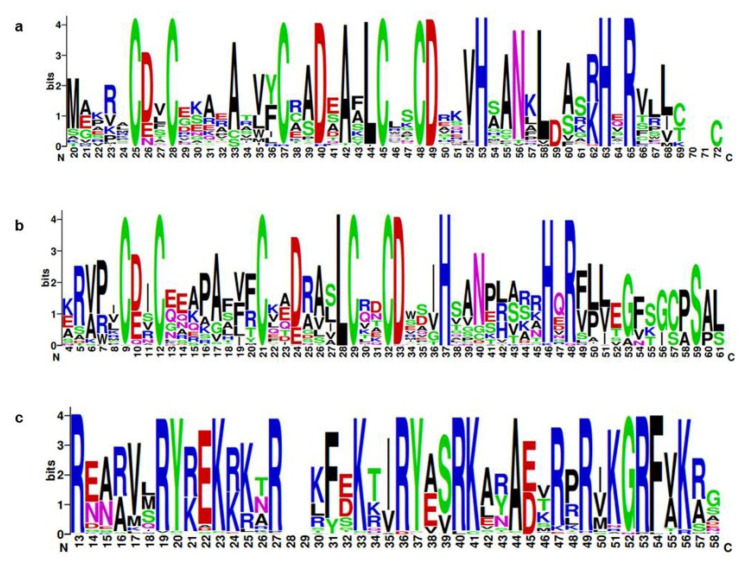
Web logos of BrBBX proteins. (**a**–**c**) Alignments of the B-box1, B-box2, and CCT domains, respectively. The *x*-axis indicates the conserved domain sequences. The height of each letter indicates how conserved the residue is across all proteins. The *y*-axis presents the relative entropy, which reflects the conservation rate of each amino acid.

The protein sequence alignment is shown in Supplementary Figure S1a–c. The protein logos were generated for all *Brassica* BBXs depicted in Figure 3. This analysis confirms the structural integrity of the domains and indicates that the B1 (Figure 3a), B2 (Figure 3b), and CCT (Figure 3c) domains were well conserved among *B. rapa*, *B. oleracea*, and *B. napus* BBX proteins.

**Figure 4:**
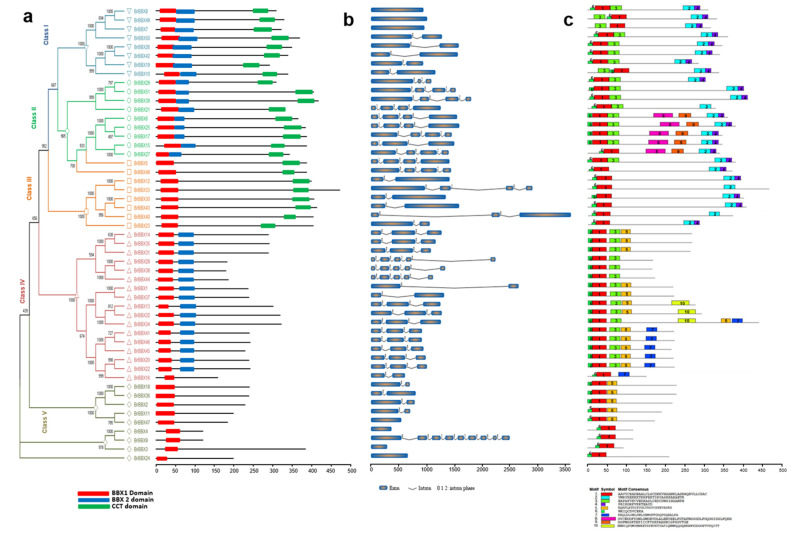
*Brassica rapa* B-box gene structures (**a**), motifs (**b**), and conserved domains (**c**). The domain architecture of the B-box genes is presented in panel a. Exons are represented by colored boxes, whereas introns are indicated by gray lines (**b**). Colored boxes indicate conserved motifs and gray lines represent non-conserved sequences. The motif lengths in each protein are presented proportionally. Classes I–V represent different groups of B-box family members in *B. rapa*.

**Figure 5:**
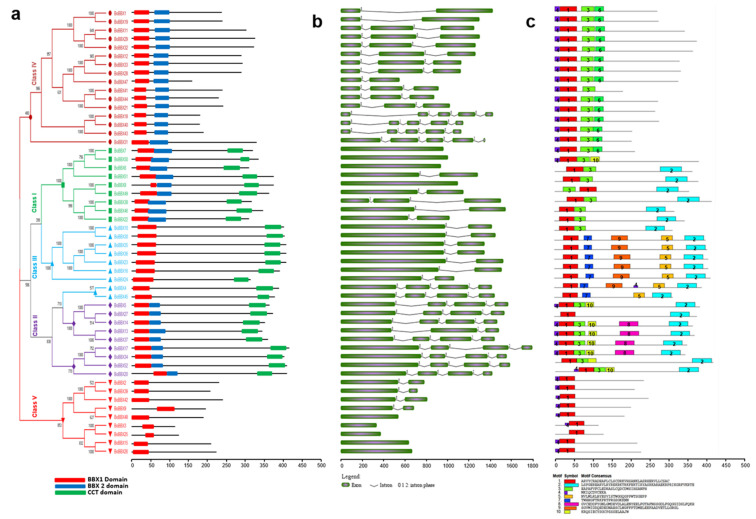
*Brassica oleracea* B-box gene structures (**a**), motifs (**b**), and conserved domains (**c**). The domain architecture of the B-box genes is presented in panel a. Exons are represented by colored boxes, whereas introns are indicated by gray lines (**b**). Colored boxes indicate conserved motifs and gray lines represent non-conserved sequences. The motif lengths in each protein are presented proportionally. Classes I–V represent different groups of B-box family members in *B. oleracea*.

**Figure 6:**
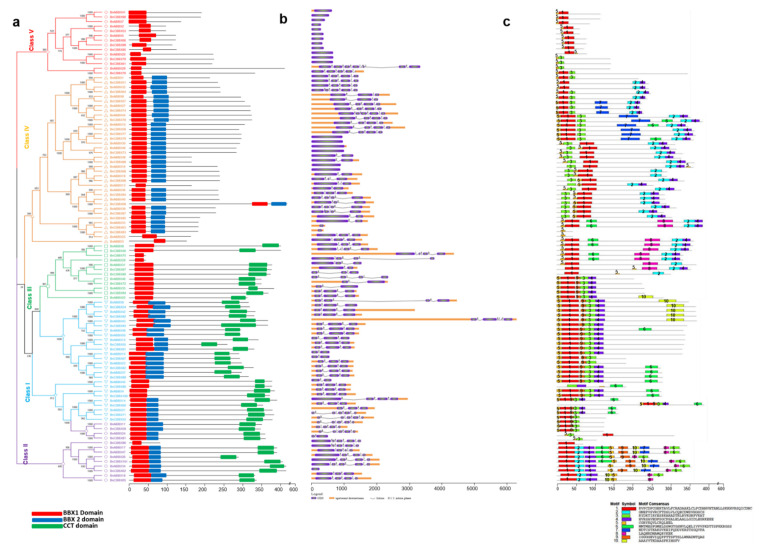
*Brassica napus* B-box gene structures (**a**), motifs (**b**), and conserved domains (**c**). The domain architecture of the B-box genes is presented in panel a. Exons are represented by colored boxes, whereas introns are indicated by gray lines (**b**). Colored boxes indicate conserved motifs and gray lines represent non-conserved sequences. The motif lengths in each protein are presented proportionally. Classes I–V represent different groups of B-box family members in *B. napus*.

For each examined *Brassica* species, the phylogenetic tree was divided into five classes (Classes I–V). In *B. rapa*, Class IV (B1 + B2) was the largest (17 BBXs). In contrast, Classes II and V comprised eleven and nine BBXs each, whereas Classes I and III included eight and nine BBXs each (Figure 4a). Similarly, in *B. oleracea*, Class IV was the largest (16 BBXs) and Class I and V contained nine BBXs, while Class II and III comprised eleven and seven BBXs (Figure 5a). Regarding *B. napus*, Class IV included 36 BBXs, which was more than the 26, 14, 13, and 12 BBXs in Classes I, V, II, and III, respectively (Figure 6a). Accordingly, Class IV (B1 + B2) was the largest for all three analyzed *Brassica* species. Class III (B1 + CCT) was the smallest class.

Different combinations of introns and exons lead to diverse gene functions. The structural diversity among the BBX genes in the three *Brassica* species was analyzed to clarify the evolution of the *Brassica* BBX gene families (Figure 4b and Figure 5b). The *B. rapa* BBX genes had 1–9 exons (Figure 4b). Most Class I genes had one or two exons, whereas the Class II genes contained three or four exons. Most of the Class III genes comprised two or four exons; the exception was *BrBBX40* (three exons). In Class IV, the majority of the genes contained three or five exons; the exceptions were *BrBBX1*, *BrBBX37*, and *BrBBX16* (two exons). In Class V, most genes harbored one or two exons, but *BrBBX9* had nine exons (Figure 4b). The *B. oleracea* BBX genes included 1–5 exons (Figure 5b). Similar to the Class I *B. rapa* genes, most of the Class I *B. oleracea* genes contained one or two exons; the exception was *BoBBX38* (three exons). In Class II, all genes harbored four exons, except for *BoBBX13* (three exons). Most of the Class III genes contained two or four exons, whereas the Class IV genes comprised 2–5 exons. In Class V, almost all genes had one or two exons, similar to the Class V *B. rapa* genes (Figure 5b). In *B. napus*, the BBX genes comprised 1–5 exons (Figure 6b). In Class I, the majority of genes contained 2–5 exons, which differed from the Class I genes in *B. rapa* and *B. oleracea* (one or two exons). Most of the Class II genes harbored 3–5 exons; the exception was *BnCBBX62* (one exon). In Class III, most genes contained 2–4 exons. The Class IV genes contained 1–5 exons. The Class V genes harbored a single exon, with the exception of *BnABBX29* (seven exons) and *BnCBBX76* (four exons) (Figure 6b). These findings reflect the structural similarity among the *Brassica* BBX genes as well as the gain and loss of exons during evolution.

We investigated the motif distribution in the BBX genes from the three examined *Brassica* species to functionally characterize the genes (Figure 4c, Figure 5c and Figure 6c). In *B. rapa*, 10 motifs were detected, of which motifs 1 and 6 were conserved (except in *BrBBX7*) (Figure 4c). In Classes I and II, almost all genes encoded motifs 1–4 and motif 6. Five of the Class II genes also included motifs 8 and 9. The Class III genes included motifs 1, 2, 4, and 6, but lacked motif 3 (except for *BrBBX29*). In Class IV, which was the largest (17 *BrBBX* genes), most of the genes contained motifs 1, 3, 5, and 6, whereas some of the genes also included motif 7 (seven *BrBBX* genes) and motif 10 (three *BrBBX* genes) (Figure 4c). In *B. oleracea*, among the 10 identified motifs, motif 1 was conserved in all 52 BBX genes (Figure 5c). Motifs 1 and 2 were common among the genes in Classes I, II, and III. In contrast, motifs 6 and 10 were, respectively, detected in only *BoBBX7* and *BoBBX50* in Class I. Motifs 8 and 10 (except in *BoBBX50*) were exclusive to the Class II genes. In Class III, most of the genes contained motifs 5, 7, and 9. In Class IV, the genes harbored motifs 1, 3, 4, and 6 (with the exception of *BoBBX41*). The Class V BBX genes encoded motifs 1 and 4 (with the exception of *BoBBX25*) (Figure 5c). The *B. napus* BBX genes included 10 motifs, of which motif 5 was conserved in all 101 genes (Figure 6c). In Class I, motifs 3, 5, and 9 were conserved in all genes, and motif 10 was detected in some of the genes. Class II contained all 10 motifs, with motifs 4 and 5 in most genes and motif 7 included in five genes. In Class III, motif 1 (except in *BnCBBX81*), motif 2 (except in five *BnBBX* genes), and motif 4 (except in five *BnBBX* genes) were conserved, whereas motif 7 was included in only four *BnBBX* genes. The 36 genes in Class IV were missing motifs 9 and 10, but motif 7 was included in nine *BnBBX* genes. In Class V, along with motif 5, we detected motif 3 in five *BnBBX* genes. Moreover, motif 1 was not detected in *BnABBX20*, *BnCBBX70*, and *BnCBBX61* (Figure 6c).

### 2.3. Chromosomal Distribution of the B. rapa, B. oleracea, and B. napus BBX Genes

We observed that 50 of 51 *BrBBX* genes, 51 of 52 *BoBBX* genes, and 79 of 101 *BnBBX* genes were randomly distributed among the chromosomes in *B. rapa* (Figure S2a), *B. oleracea* (Figure S3a), and *B. napus* (Figure S4a), respectively. One *B. rapa* gene (*BrBBX51*), one *B. oleracea* gene (*BoBBX52*), and 22 *B. napus* genes were not mapped on any chromosome. In *B. rapa*, chromosome A02 harbored the most *BrBBX* genes (eight), followed by chromosomes A01 and A07, both of which had seven *BrBBX* genes (Figure S2a). Chromosome A04 contained the fewest *BrBBX* genes (two). The *BrBBX* genes were mapped on all 10 chromosomes, reflecting their broad chromosomal distribution. In *B. oleracea*, chromosomes C02 and C07 had the most *BoBBX* genes (seven), followed by chromosomes C01, C03, C06, and C09, which contained six *BoBBX* genes (Figure S3a). Similar to *B. rapa*, chromosome C04 in *B. oleracea* had the fewest *BoBBX* genes (three). The *B. napus BnBBX* genes were mapped on the AA and CC genomes. In the A genome, chromosome A02 contained the most *BnBBX* genes (seven) (similar to *B. rapa* and *B. oleracea*), followed by chromosomes A07 (six) and A08 (five) (Figure S4a). Similar to *B. rapa* and *B. oleracea*, chromosome A04 had the fewest *BnBBX* genes (three). Three or four genes were clustered at the end of chromosomes A04, A06, and A08. In the C genome, chromosome C06 contained the most *BnCBBX* genes (seven), followed by chromosomes C02 and C09, which had six *BnBBX* genes (Figure S4a). Unlike *B. rapa* and *B. oleracea*, the fewest *BnBBX* genes (three) were mapped on chromosome C01 (Figure S4a). The distribution analysis indicated the BBX genes were most abundant on chromosome 2 in all examined *Brassica* genomes, possibly because of gene duplication events.

### 2.4. Analyses of Synteny and Duplication of BBX Orthologous Genes

Whole-genome and tandem duplications are critical events for enhancing genome complexity and the evolution of novel gene functions. Only one gene pair (single tandem array) was associated with a tandem duplication in the *B. rapa*, *B. oleracea*, and *B. napus* genomes (Table S4). Single tandem duplication events were detected in *B. rapa* (chromosome A10) involving *Bra008668* and *Bra008669* as well as in *B. oleracea* (chromosome C09) involving *Bo9g163720.1* and *Bo9g163730.1*. In *B. napus*, *BnaC09g41980D* and *BnaC09g41990D* on chromosome C09 were the result of a single duplication event (Table S4).

Thirty gene pairs in *B. rapa* (Figure S2b; Table S5), 27 gene pairs in *B. oleracea* (Figure S3b; Table S6), and 64 gene pairs in *B. napus* (Figure S4b; Table S7) were segmentally duplicated. Our analysis also revealed the variability in the number of duplicated genes per segmental duplication event and an uneven distribution of these duplications on seven of 10 chromosomes in *B. rapa*, six of nine chromosomes in *B. oleracea*, and 17 of 19 chromosomes in *B. napus* (Tables S5–S7). In *B. rapa*, chromosomes A01 and A02 had the most gene pairs (nine and eight, respectively), whereas chromosomes A06 and A09 contained the fewest gene pairs (one each). In *B. oleracea*, chromosomes C02 and C08, respectively, had the most (nine) and fewest (one) gene pairs. In *B. napus*, chromosomes A02 and A08 contained the most gene pairs (10 and 11, respectively), whereas chromosomes A01, C01, C03, C05, C07, and C08 had the fewest gene pairs (one each). Additionally, the *B. napus* A genome included more segmentally duplicated gene pairs than the C genome (Tables S5–S7).

### 2.5. Comparative Synteny Analysis of Orthologous Pairs of BBX Genes in Brassicaceae

**Figure 7:**
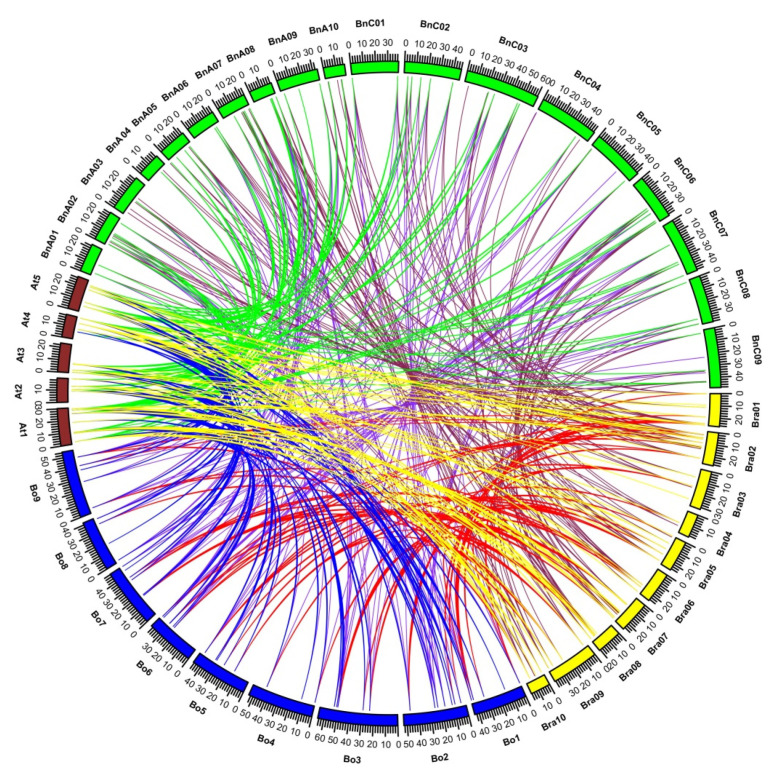
*Brassica rapa* (Bra01–Bra10), *B. oleracea* (C01–C09), *B. napus* (Bna01–Bna10 and Bnc01–Bnc09), and *Arabidopsis thaliana* (At1–At5) chromosomal maps were constructed according to orthologous gene pair positions. The maps revealed highly conserved synteny. The orange, blue, and red curves link the BBX genes on *A. thaliana* chromosomes 1, 2, 3, 4, and 5 with their orthologous genes in *B. rapa*, *B. oleracea*, and *B. napus*, respectively.

In addition to identifying gene duplication events, we investigated the synteny between the BBX gene families of *A. thaliana*, *B. rapa*, *B. oleracea*, and *B. napus.* A total of 43 chromosomes (5, 10, 9, and 19 from *A. thaliana*, *B. rapa*, *B. oleracea*, and *B. napus*, respectively) and 236 BBX genes (32, 51, 52, and 101 from *A. thaliana*, *B. rapa*, *B. oleracea*, and *B. napus*, respectively) were used to investigate syntenic relationships. Orthologous BBX genes and genes resulting from segmental duplications are indicated by different colored lines in Figure 7. The *BrBBX* genes on chromosomes 1 (BrA01), 2 (BrA02), and 10 (BrA10) had orthologs on chromosomes AT1, AT3, AT4, and AT5 (*A. thaliana*), C01, C02, and C09 (*B. oleracea*), and A01, A02, A08, A09, A10, C01, C02, C06, C08, and C09 (*B. napus*). A total of 235 BBX gene pairs (Tables S8–S13) had syntenic relationships between *A. thaliana* and *B. rapa*, *B. oleracea*, and *B. napus*. Additionally, 58 orthologous pairs were detected between *A. thaliana* and *B. rapa* as well as between *A. thaliana* and *B. oleracea*, which was fewer than the 68 orthologous pairs detected between *A. thaliana* and *B. napus*. Fifty-six orthologous pairs were identified between *B. rapa* and *B. oleracea* as well as 121 orthologous pairs between *B. rapa* and *B. napus* and 123 orthologous pairs between *B. oleracea* and *B. napus* (Tables S8–S13).

Of the 58 orthologous gene pairs between *A. thaliana* and *B. rapa*, eight *AtBBX* genes had a single copy of an orthologous gene in *B. rapa*. In contrast, there were 2–4 copies of the other *AtBBX* orthologs in *B. rapa* (Table S8). More specifically, there were two copies of seven *AtBBX* orthologs, three copies of nine *AtBBX* orthologs, four copies of the *AtBBX24* ortholog, and five copies of the *AtBBX28* ortholog in *B. rapa*. An analysis of the synteny among orthologs revealed the genes generally belonged to the same groups. For example, *AtBBX1* (Group I) was syntenic with the Group I genes *BrBBX7*, *BrBBX8*, and *BrBBX49*. However, there were some exceptions. Specifically, *AtBBX9* and *AtBBX10*, which belong to Group II (B1 + B2 + CCT), had syntenic relationships with *BrBBX5*, which belongs to Group III (B1 + CCT), indicative of a loss of the B2 domain during evolution. Altogether, 26 *A. thaliana* BBX genes were syntenic with 46 *B. rapa* genes (Table S8).

An examination of the 58 orthologous gene pairs between *A. thaliana* and *B. oleracea* indicated that the *B. oleracea* genome included single copies of eight *AtBBX* orthologs (Table S9). Interestingly, there were single copies of the *AtBBX4*, *AtBBX9*, *AtBBX10*, *AtBBX11*, and *AtBBX17* orthologs in both *B. rapa* and *B. oleracea*, reflecting their identical genomic arrangements. Among the other orthologous gene pairs, there were two copies of 12 *AtBBX* orthologs (segmental duplications), three copies of six *AtBBX* orthologs, and four copies of *AtBBX15* and *AtBBX28* orthologs in *B. oleracea* (Table S9). Moreover, the orthologous gene pairs belonged to the same groups (according to their domains), with the exception of the *AtBBX9* and *AtBBX10* orthologs in *B. oleracea*, which lacked the B2 domain. A comparative analysis of *A. thaliana* and *B. napus* indicated that of the 68 orthologous gene pairs, there was a single copy of four *AtBBX* orthologs, two and four copies of six *AtBBX* orthologs, and three copies of three *AtBBX* orthologs in *B. napus* (Table S10). Although *AtBBX9* and *AtBBX10* orthologs were detected in *B. rapa* and *B. oleracea*, they were missing in *B. napus.*

We detected 56 orthologous gene pairs between *B. rapa* and *B. oleracea*. A single copy of nine *BrBBX* orthologs, two copies of 13 *BrBBX* orthologs, and three copies of seven *BrBBX* orthologs were detected in *B. oleracea* (Table S11). A total of 29 *B. rapa* genes had syntenic relationships with 28 *B. oleracea* genes. The *BrBBX6* gene, which belongs to Group II (B1 + B2 + CCT), was syntenic with *BnABBX14*, *BnABBX21*, *BnCBBX52*, *BnCBBX60*, and *BnCBBX71*, which belong to Group I (Tables S11–S13). Of the 121 orthologous gene pairs between *B. rapa* and *B. napus*, there was a single copy of seven *BrBBX* orthologs, two copies of 11 *BrBBX* orthologs, as well as three, four, and five copies of 9, 10, and 5 *BrBBX* orthologs, respectively, in *B. napus* (Table S12). Moreover, *BrBBX9* was syntenic with the exostosin genes in *B. napus* (*BnaA03g06300D*, *BnaA10g17600D*, *BnaC03g08100D*, and *BnaC09g40970D*), whereas *BrBBX10*, *BrBBX24*, *BrBBX33*, and *BrBBX48* (Table S12) were syntenic with the *B. napus* CCT superfamily genes (*BnaA10g11600D*, *BnaA03g36070D*, *BnaA07g24620D*, *BnaA10g03390D*, and *BnaC05g03370D*), which included only the CCT domain (Table S12). Overall, 42 *B. rapa* BBX genes were syntenic with 71 *B. napus* genes.

**Figure 8:**
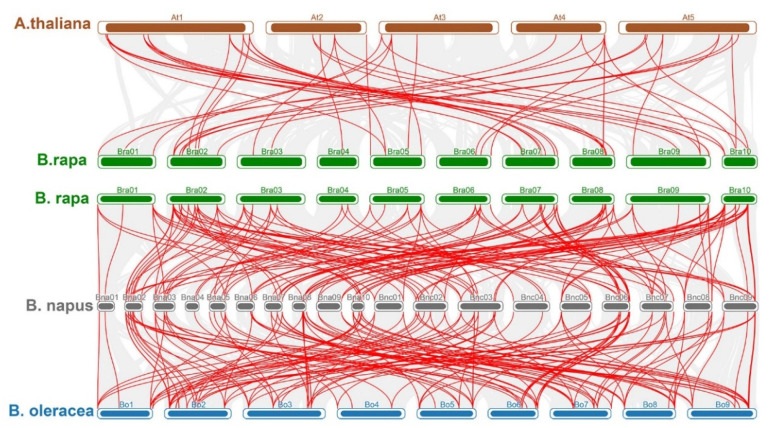
Syntenic collinearity relationship of B-box genes among *A. thaliana* and *B. rapa*, *B. oleracea*, and *B. napus*. In the background, gray lines designate the collinear blocks within the *A. thaliana* and other Brassica genomes. Diverse colored lines indicate the relationships among orthologous gene pairs between four Brassica species.

A comparative analysis of the common orthologous pairs of BBX genes among four species (*B. rapa*, *A. thaliana*, *B. oleracea*, and *B. napus*) identified orthologs of 26 *B. rapa* BBX genes in the *A. thaliana*, *B. oleracea*, and *B. napus* genomes (Figure 8; Table S14). Almost all of the BBX genes had a one-to-one relationship between the *B. rapa* and *A. thaliana* genomes; the exceptions were the *Bra008668*, *Bra010994*, *Bra023541*, and *Bra031180* orthologs in *A. thaliana* (two copies each). However, the comparison with *B. oleracea* indicated there were two or three copies of most BBX genes. Only six BBX genes were detected as single copies. Furthermore, there were multiple copies (between two and five) of almost all *B. rapa* and *A. thaliana* BBX orthologs in *B. napus*. The exception was the *Bra004035* ortholog (single copy).

### 2.6. Analysis of the Ka and Ks Values of the Orthologous BBX Genes among Brassica Species and Arabidopsis Thaliana

The non-synonymous mutation rate (Ka) and the synonymous mutation rate (Ks) were calculated to determine the selection pressure associated with the duplication of BBX genes in *Brassica* species (Tables S8–S10). First, the orthologous BBX genes among *A. thaliana* and the three *Brassica* species were identified. The 58, 56, and 121 pairs of orthologous BBX genes between *A. thaliana* and *B. rapa*, *B. oleracea*, and *B. napus*, respectively, were used to calculate the Ka, Ks, and Ka/Ks values (Tables S8–S10). Most of the Ka/Ks values for the *Brassica* species were less than 1, indicating the orthologous genes were under strong purifying selection pressure. The Ka/Ks value exceeded 1 for one orthologous gene pair between *B. rapa* and *B. napus* (*Bra003748* and *BnaA07g21640D*), reflecting positive selection pressure.

### 2.7. Cis-Elements in the Brassica BBX Gene Promoters

Cis-acting elements in the promoter region are crucial for regulating the expression of the corresponding gene because they bind with various transcription factors [50]. Thus, we identified the cis-regulatory elements in 1500-bp sequences upstream of the start codon of genes in *B. rapa*, *B. oleracea*, and *B. napus* using the PlantCARE database (Table S15). As predicted, common promoter elements (e.g., TATA-box and CAAT-box) were identified in all *Brassica* BBX gene promoters. Several cis-elements related to plant development and growth, phytohormone responses, light responses, and stress responses were detected in all three *Brassica* species. The following 15 light-responsive cis-elements involved in growth and development were detected in the BBX gene promoter regions: ARE, G-box, ABRE, Box4, GT1-motif, AE box, MRE, ACE, I-box, chs-CMA, GA motif, GAP-box, 3-AF1 binding site, Sp1, and MSA-like (Table S15). Among the cis-acting elements involved in hormone responses, ABRE, ERE, GAREs (GARE-motif and P-box), and the MeJA-responsive elements (CGTCA-motif and TGACG-motif) were, respectively, identified in the promoter regions of 41, 31, 27, and 62 BBX genes in *B. rapa*, 42, 28, 32, and 81 BBX genes in *B. oleracea*, and 86, 55, 64, and 143 BBX genes in *B. napus*. The zein metabolism regulation element (O2 site) was detected in 12 *BrBBX*, 21 *BoBBX*, and 31 *BnBBX* genes, whereas the CAT-box influencing meristem expression was identified in the promoter regions of 18 *BrBBX*, 22 *BoBBX*, and 39 *BnBBX* genes.

Of the stress-related response elements, ARE, which is an anaerobic induction element, was detected in the promoters of most BBX genes. Some stress-related (low temperatures, wounding, and drought) cis-acting elements were also identified in the promoter regions of BBX genes (Table S15).

### 2.8. Developmental and Tissue-Specific Expression of BrBBX Genes

**Figure 9:**
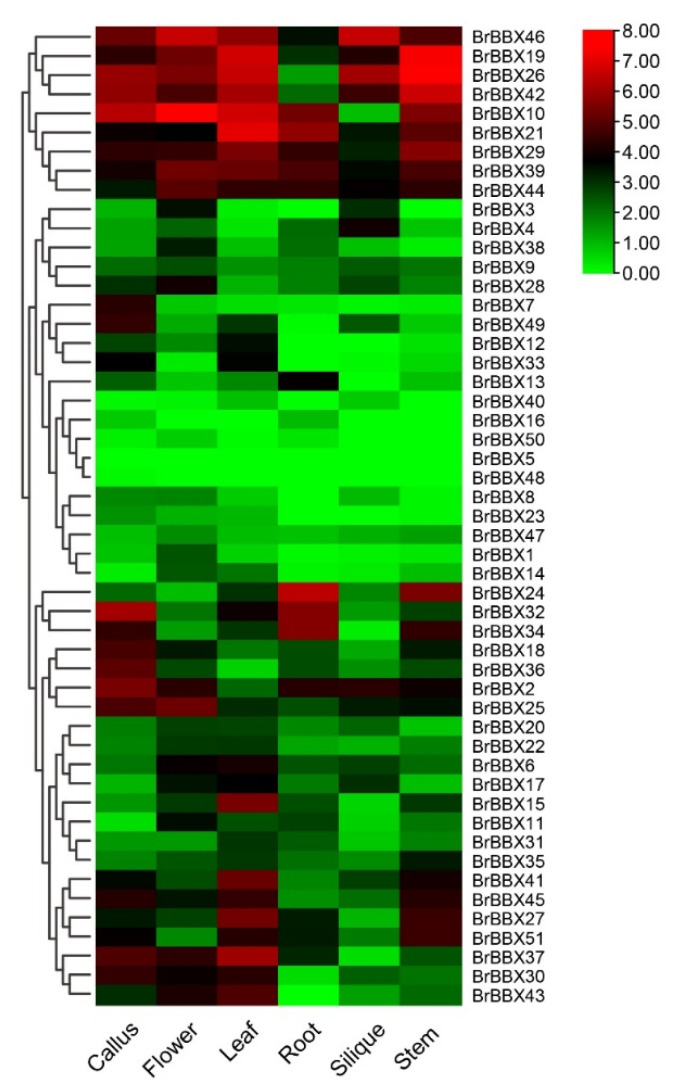
Tissue-specific *BrBBX* expression patterns. The relative expression data were log_2_-transformed using the pheatmap package of R. A cluster dendrogram is provided to the left of the heat map. Blue and red indicate downregulated and upregulated expression, respectively.

We investigated the expression patterns of 51 *BrBBX* genes in different organs and during various developmental stages using available transcriptome data [51]. Gene expression was analyzed in the callus, flower, leaf, root, silique, and stem. The expression levels varied among genes, with some genes exhibiting tissue-specific expression (Figure 9). These results suggest that BBXs in *B. rapa* may affect diverse biological processes in different tissues. Of the 51 *BrBBX* genes, 12 (*BrBBX19*, *BrBBX21*, *BrBBX25*, *BrBBX29*, *BrBBX6*, *BrBBX35*, *BrBBX39*, *BrBBX41*, *BrBBX44*, *BrBBX45*, *BrBBX46*, and *BrBBX51*) were relatively highly expressed in all developmental stages and tissues, reflecting their overall involvement in *B. rapa* plant development. Some BBX genes (*BrBBX5*, *BrBBX16*, *BrBBX40*, *BrBBX48*, and *BrBBX51*) were expressed at very low levels in all organs in different growth stages. Additionally, *BrBBX27*, *BrBBX32*, and *BrBBX37* were expressed at higher levels in the silique than in the stem, flower, leaf, and root (Figure 9). 

Similarly, *BrBBX26*, *BrBBX30*, and *BrBBX43* were expressed exclusively in the root, whereas *BrBBX7* was expressed only in the callus. The *BrBBX10* gene was highly expressed in all organs, except for the silique, and *BrBBX19*, *BrBBX21*, and *BrBBX26* were expressed at high levels in all organs. The other BBX genes were expressed at lower levels in the various organs. Moreover, the genes in the same group had identical expression patterns.

### 2.9. BrBBX Expression Patterns in Response to Various Abiotic Stresses and Hormones

**Figure 10:**
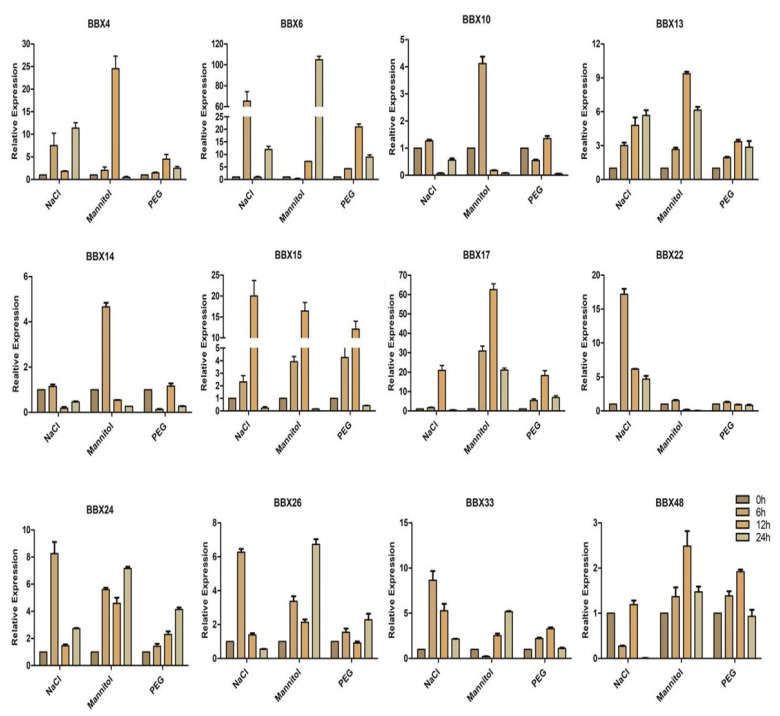
Abiotic stress-induced *BrBBX* expression profiles. The graphs present the expression levels in *Brassica* plants following NaCl, mannitol, and PEG treatments. Samples were collected at various time points for a qRT-PCR analysis. The *x*-axis presents the abiotic stresses. The *y*-axis presents the relative expression levels. Error bars indicate the standard deviations of three independent qRT-PCR biological replicates.

**Figure 11:**
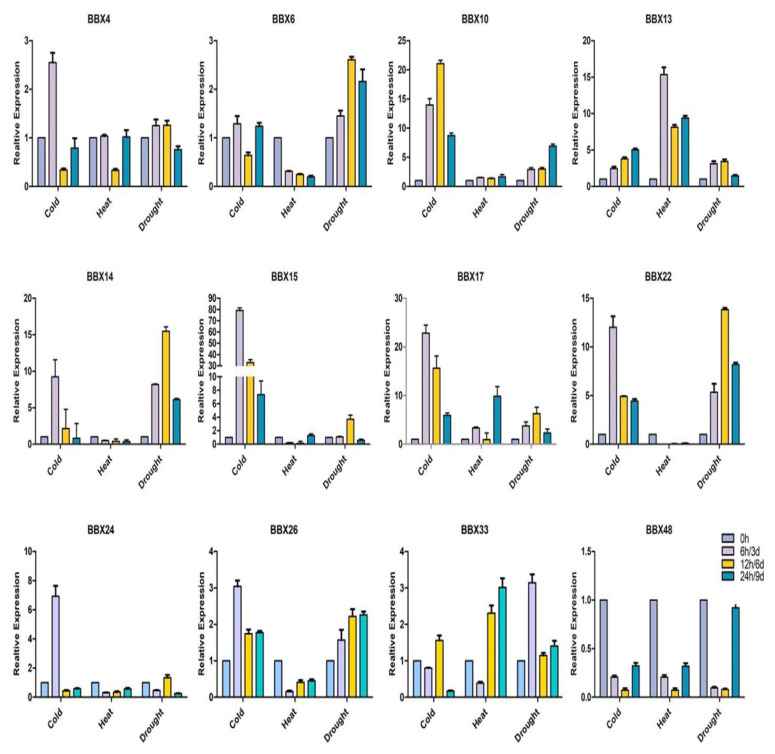
Inducible expression profiles of *Brassica* BBX genes in response to cold, heat, and drought conditions. The *x*-axis presents the treatments. The *y*-axis presents the expression levels relative to the expression at the 0-h time point. Error bars indicate the standard deviations of three independent qRT-PCR biological replicates.

**Figure 12:**
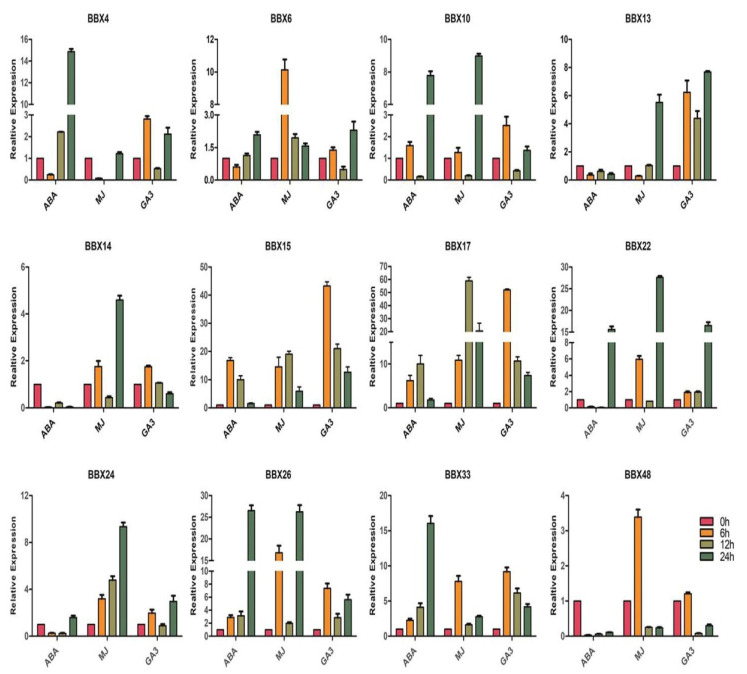
Inducible expression profiles of *Brassica* BBX genes in response to exogenously applied hormones. The *x*-axis presents the treatments. The *y*-axis presents the expression levels relative to the expression at the 0-h time point. Error bars indicate the standard deviations of three independent qRT-PCR biological replicates.

Abiotic stresses and hormones substantially influence plant development and growth. To clarify the effects of different abiotic stresses and hormones on the expression of *Brassica* BBX genes, the BBX gene expression patterns at various time points were analyzed by qRT-PCR. We randomly selected 12 *BrBBX* genes (*BrBBX4*, *BrBBX6*, *BrBBX10*, *BrBBX13*, *BrBBX14*, *BrBBX15*, *BrBBX17*, *BrBBX22*, *BrBBX24*, *BrBBX26*, *BrBBX33*, and *BrBBX48*) from five groups and examined their expression following an exposure to different abiotic stresses and hormones (i.e., NaCl, mannitol, PEG, cold, heat, drought, ABA, SA, and MeJA). The *BrBBX15* and *BrBBX17* expression levels were upregulated by all abiotic stresses and exogenous hormone treatments (i.e., compared with the samples at 0 h) (Figure 10, Figure 11 and Figure 12). The most upregulated gene was *BrBBX6* at 24 h after the mannitol treatment (104-fold increase). Additionally, *BrBBX6* was also highly expressed in response to NaCl (6 h) and PEG (12 h) (65-fold and 21-fold increases, respectively). The qRT-PCR analysis indicated that *BrBBX* expression levels were affected by NaCl, mannitol, and cold stress more than by PEG, heat, and drought (Figure 10 and Figure 11). Exposure to heat stress generally downregulated the expression of *BrBBX* genes, with the exception of *BrBBX13*, *BrBBX15*, *BrBBX17*, and *BrBBX33*.

We observed that *BrBBX10*, *BrBBX22*, *BrBBX26*, and *BrBBX33* were highly expressed at some time points following the ABA, MeJA, and GA3 treatments (Figure 12). The *BrBBX14*, *BrBBX24*, and *BrBBX48* expression levels were upregulated by the application of exogenous MeJA, but were relatively unaffected by ABA and GA3. Interestingly, among the 12 selected *BrBBX* genes, only *BrBBX4* was expressed at very low levels after the exogenous MeJA treatment. Regarding the effects of the exogenous ABA treatment, *BrBBX15*, *BrBBX17*, *BrBBX26*, and *BrBBX33* expression levels were upregulated (minimum 0.5-fold increase) at all time points. Moreover, *BrBBX6*, *BrBBX15*, *BrBBX17*, *BrBBX24*, and *BrBBX26* were also highly expressed at all time points after the MeJA treatment. For both ABA and MeJA, the *BrBBX* genes were most highly expressed at the 24-h time point. Regarding the effects of GA3, *BrBBX13*, *BrBBX15*, *BrBBX17*, *BrBBX22*, *BrBBX26*, and *BrBBX33* expression levels were upregulated at almost all time points, but especially at 6 h post-treatment (Figure 12).

## 3. Discussion

*BBX* proteins have recently been confirmed as important transcription factors with vital regulatory roles affecting the economic value of *Brassica* species, which are important oilseed and vegetable crops worldwide; thus, the *BBX* gene families in *B. rapa*, *B. napus*, and *B. oleracea* should be comprehensively investigated. A few *Brassica* species are susceptible to numerous abiotic factors (e.g., drought, salinity, and temperature extremes) and biotic factors (e.g., bacteria, fungi, insect pests, and viruses) [41]. In the present study, we identified *BBX* genes in three *Brassica* species and investigated their phylogenetic relationships, linkage group organization, intron–exon organization, duplication events, conserved motifs, cis-acting elements, and expression patterns in different tissues following various abiotic stress treatments, which is summarized in Table S16. The global identification of *Brassica BBX* genes provides the foundation for advanced functional studies of these genes in Brassicaceae crops.

### 3.1. Identification and Evolution of Brassica BBX Genes

In this study, we identified 51, 52, and 101 *BBX* genes in *B. rapa*, *B. oleracea*, and *B. napus*, respectively. The *BBX* gene families are larger in these three *Brassica* species than in other plant species, including *A. thaliana* (32 *BBX* genes) [4], rice (30 *BBX* genes) [9], pear (25 *BBX* genes) [45], tomato (29 *BBX* genes) [31], potato (30 *BBX* genes) [47], apple (64 *BBX* genes) [32], and sweet cherry (15 *BBX* genes) [52]. To date, the 101 *BnBBX* genes represent the largest *BBX* gene family among plant species. These results suggest that the *BBX* gene families in *Brassica* species, including *B. napus*, expanded considerably during speciation. Gene and whole-genome duplication events occurred approximately 35 million years ago in all Brassicaceae genomes [53,54]. Moreover, *Brassica* genomes underwent a lineage-specific whole-genome triplication about 15.9 million years ago [55,56], which may have contributed to the diversification and expansion of the *Brassica* *BBX* gene families. Additionally, in the case of *B. napus*, an allopolyploidization followed by the fusion of genomes A and C may have been critical for increasing the number of *BnBBX* genes [57].

During plant evolution, segmental and tandem duplication events are crucial for expanding gene families [58]. We analyzed the segmental and tandem duplications in *Brassica* species, which revealed 30, 27, and 64 pairs of BBX genes in *B. rapa*, *B. oleracea*, and *B. napus*, respectively, that were derived from segmental duplication events (Tables S5–S7). However, only *BrBBX49*/*BrBBX50* in *B. rapa*, *BoBBX50*/*BoBBX51* in *B. oleracea*, and *BnBBX92*/*BnBBX93* in *B. napus* were identified as tandem duplicates (Table S4). Accordingly, segmental duplications were the major contributor to the expansion of the *Brassica BBX* families during evolution. The fatty acid desaturase and heat stress transcription factor gene families in *Brassica* species likely evolved similarly [59,60]. In the current study, the Ka/Ks values of the segmentally duplicated gene pairs were calculated. Usually, Ka/Ks values less than and greater than 1 signify negative and positive selection pressures, respectively. A Ka/Ks value equal to 1 reflects neutral selection. The Ka/Ks values for the *BBX* gene pairs were less than 1 in all three *Brassica* species, with the exception of one gene pair in *B. oleracea* and *B. napus*. Hence, most of the *Brassica* *BBX* gene pairs evolved under purifying selection. This is consistent with the purifying selection reported for the apple [32] and grapevine [33] *BBX* gene families. The purifying selection pressure may have helped to maintain the conserved structures of the *BBX* genes during evolution.

*Brassica* genomes appear to contain more *BBX* genes than the genomes of other plant species, including tomato and potato. However, *Brassica* species have smaller genomes, i.e., 529 Mb in *B. rapa* [55], 696 Mb in *B. oleracea* [61], and approximately 1.2 Gb in *B. napus* [62], than tomato (960 Mb) [63] and potato (840 Mb) [64]. These findings suggest that the number of *BBX* genes is not directly related to plant genome size. Moreover, although there were no significant differences in the number of *BBX* genes among plant species, the *BBX* gene types varied. For example, the potato genome contains nine, nine, five, and seven *BBX* genes in Classes I, IV, III, and V, respectively [47]. In contrast, the tomato genome includes 8, 10, 5, and 6 *BBX* genes in Classes I, IV, III, and V, respectively [31]. In *Brassica* species, the number of genes in these classes is the same in the diploid genomes (i.e., 18, 16, 9, and 9 *BBX* genes in Classes I, IV, III, and V, respectively, in both *B. rapa* and *B. oleracea*), whereas the allotetraploid amphidiploid genome of *B. napus* contains 34, 20, 33, and 14 *BBX* genes in Classes I, IV, III, and V, respectively.

On the basis of the phylogenetic and sequence alignment analyses, the *Brassica BBX* family members were classified into five subgroups, similar to the *BBX*s in *A. thaliana*, tomato, pear, and potato [4,31,45,46,47]. The *Brassica BBX* family members from Groups I, II, and IV contained the B1 and B2 domains, with the exception of *BrBBX5*, *BrBBX48*, *BoBBX4*, and *BoBBX45*, which should belong to Group III (B1 + CCT), but were in Group II instead (B1 + B2 + CCT) (Figure 2). In contrast to animal *BBX*s with two different *BBX* domains, the amino acid sequences of the two BBX domains in *Brassica* species are highly conserved and have the same topology [7]. Most of the BBXs in green algae reportedly only have a single BBX domain, but in the unicellular green alga *Chlamydomonas reinhardtii*, two BBX domains have been detected in BBXs. These observations imply that *BBX* gene duplication events occurred in some circumstances well before plants colonized land [3,6,65]. During evolution, *BBX* gene families expanded rapidly. Furthermore, the highly conserved nature of BBX proteins across the plant kingdom suggests these proteins may have played a significant role in the development of terrestrial plants [3,65].

### 3.2. Potential Roles of BrBBX Genes Related to Plant Growth and Development

Previous studies concluded that *BBX* genes are important for diverse plant developmental and growth processes, including shade avoidance, seedling photomorphogenesis (e.g., hypocotyl growth), chlorophyll accumulation, and flowering [3,66,67]. In the current study, various cis-acting regulatory elements were identified in the promoters of *Brassica BBX* genes. Light-responsive cis-regulatory elements were commonly detected in the *Brassica BBX* gene promoter regions. More specifically, in *B. rapa*, *B. oleracea*, and *B. napus*, the following light-responsive elements were identified: ARE, G-box, Box4, GT1-motif, AE box, MRE, I-box, chs-CMA, GA motif, GAP-box, 3-AF1 binding site, Sp1, and MSA-like. The circadian cis-element and the GATA cis-element, which are involved in light-regulated and tissue-regulated expression, were identified in the promoter region of 22 *B. rapa*, 28 *B. oleracea*, and 43 *B. napus BBX* genes. These results suggest that the *Brassica* *BBX* genes may be functionally similar to *A. thaliana AtBBX* genes, which are under the control of the circadian rhythm and are mostly involved in light-related processes, including flowering, photomorphogenesis, and shade avoidance [3]. In *A. thaliana*, the overexpression of *AtBBX6* (*COL5*) induces early flowering by upregulating *FT* expression, whereas the overexpression of *AtBBX7* (*COL9*) delays flowering under short-day conditions because of the associated repressed expression of *FT* and *CO* [15,68]. Previous research indicated that *AtBBX32*/EIP6 in *A. thaliana* regulates the flowering time apparently in a CO-independent manner under long-day conditions [69]. In the current study, *BrBBX6* and *BrBBX10*, which are, respectively, homologs of *AtBBX7* and *AtBBX6*, were highly expressed in all examined tissues, except for the silique in the case of *BrBBX10*. Both genes were expressed at high levels in floral tissues, indicative of a potential regulatory role related to flower development. Additionally, *AtBBX21* (i.e., SALT TOLERANCE HOMOLOG 2) positively regulates seedling photomorphogenesis after interacting with HY5, which is a key regulator of photomorphogenesis [66]. Thus, *AtBBX21* helps control seed germination and ABA signaling. Moreover, *AtBBX21* is expressed at relatively high levels in germinating seeds and dry seeds [70]. In the present study, *BrBBX32* and *BrBBX34*, which are the closest homologs of *AtBBX21*, were expressed at lower levels in the silique than in all other analyzed tissues. In contrast to *AtBBX21* (STH2), both *AtBBX24* (STO) and *AtBBX25* (STH) suppress seedling photomorphogenesis [19,71]. We observed that *BrBBX46*, which is closely related to *AtBBX24*, was most highly expressed in the silique and flower. Hence, *BrBBX*s might be crucial for *B. rapa* photomorphogenesis. The *BrBBX46* gene was expressed in diverse tissues, including the flower, leaf, root, and stem. Accordingly, *BrBBX46* might have diverse regulatory functions influencing plant development and growth. Considered together, our results suggest that *Brassica BBX* family members are involved in floral development, while also contributing to seedling photomorphogenesis. They appear to have multiple significant roles in various biological, growth, and developmental processes.

### 3.3. Potential Roles of BrBBX Genes in Response to Abiotic Stresses and Exogenous Hormones

Environmental conditions (e.g., drought, salinity, cold, and heat) affect plant growth and development and limit plant productivity [72,73]. Various stresses modulate plant development and growth and modify transcription in plants by affecting specific proteins and post-translational modifications as well as by altering signaling pathways involving many genes [74]. In this study, we proved that *Brassica BBX* genes are highly responsive to various simulated stresses and hormones, including drought, mannitol, PEG, salt, cold, heat, ABA, GA3, and MeJA, implying *BBX* genes contribute to multiple stress responses in *Brassica* species. Earlier studies demonstrated the involvement of plant *BBX* genes in responses to different abiotic stresses and hormone treatments [33,49]. More specifically, the salt tolerance protein STO (*AtBBX24*) is activated by salt stress in yeast cells [34]. It also increases the root length of *A. thaliana* plants exposed to salinity stress [35]. Additionally, *AtBBX18* negatively regulates photomorphogenesis and thermotolerance in *A. thaliana*, while also negatively regulating the transcription of heat-responsive genes, such as *APX2*, *DGD1*, *Hsp70*, and *Hsp101*, thereby decreasing the seed germination and seedling survival rates after heat treatment [39]. In chrysanthemum, *CmBBX24* mediates drought and cold tolerance and delays flowering [9]. The constitutive expression of *CmBBX22* in transgenic *A. thaliana* leads to enhanced drought tolerance [75]. The *BrBBX14* gene, which is a close homolog of *CmBBX22*, is highly expressed in *Brassica* species under drought conditions [75]. Furthermore, various studies confirmed that *BBX* genes affect hormone signaling pathways. A study on *A. thaliana* determined that *BBX* gene expression is altered by cyclic ADP-ribose (cADPR) at different temperatures and by ABA treatments [76,77]. In pea, BBXs modulate and regulate the COP/HY5 signaling pathway, with *BBX18* also possibly affecting GA pathways [78]. In this study, we detected at least one stress-responsive cis-element (e.g., ARE, ABRE, MBS, TC-rich, and LTR) in the promoter region of *Brassica* *BBX* genes, suggesting the encoded proteins have significant roles in abiotic stress responses (Table S15). Furthermore, the cold treatment substantially affected the expression of at least six genes (*BrBBX10*, *BrBBX14*, *BrBBX15*, *BrBBX17*, *BrBBX22*, and *BrBBX24*), whereas the heat treatment modified the expression of two genes (*BrBBX13* and *BrBBX17*) (Figure 11). The TC-rich and LTR cis-elements were detected in the promoter region of these genes. Eight genes (*BrBBX6*, *BrBBX10*, *BrBBX13*, *BrBBX14*, *BrBBX15*, *BrBBX17*, *BrBBX22*, and *BrBBX24*) that were expressed at high levels following the drought, mannitol, and PEG treatments have MBS or ABRE elements in their promoter region, with the exception of *BrBBX17*. Our data indicate that eight *Brassica BBX* genes highly expressed in response to salt stress have promoters with ABRE, MBS, MYC, or MYB elements. We observed that salt, osmotic, and cold stresses upregulated the expression of most of the *Brassica BBX* genes, whereas drought and PEG only moderately increased expression and the heat treatment downregulated expression (Figure 10 and Figure 11). These results indicate that the expression of most of the *BrBBX* genes was induced or repressed to varying degrees depending on the type of stress. Some genes, such as *BrBBX15* and *BrBBX17* (Figure 10 and Figure 11), were highly expressed in response to all abiotic stresses, suggesting these genes might contribute to responses to multiple stress signals. The *BrBBX6* gene was highly expressed following the NaCl, mannitol, and PEG treatments, but its expression was downregulated by cold and heat stress, implying that *BrBBX6* might influence tolerance to only specific abiotic stresses. In this study, *Brassica BBX* gene expression was affected by the application of different hormones (Figure 12). The *BrBBX15*, *BrBBX17*, *BrBBX22*, *BrBBX26*, and *BrBBX33* expression levels were substantially upregulated by ABA, MeJA, and GA3. Most of the *BrBBX* genes were responsive to ABA, MeJA, and GA3 at different time points, but gene expression was affected more by MeJA and GA3 than by ABA. This indicates that these genes might regulate various aspects of the *Brassica* hormone signaling pathways. Only *BrBBX48* was repressed or unaffected in all treatments. Additionally, we investigated the already characterized B-Box genes in different plant species and found that most of the genes belonged to Group IV (B-box1 + B-box2) and Group V (B-box1) (Table S18). This indicates that Group IV and V are relatively well characterized compared to the rest of the groups for abiotic and hormonal stress. Similarly, in our study, *BrBBX13, BrBBX14, BrBBX22* (all three from Group IV) as well as *BrBBX4* and *BrBBX24* (Group V) are highly expressed in abiotic (heat, cold, salt, and drought) and hormonal (ABA, MeJA, and GA3) stress. 

## 4. Materials and Methods

### 4.1. Identification of BBX Family Members in Brassica Species

To identify and annotate *BBX* genes in *B. rapa*, previously reported *A. thaliana* BBX protein sequences were downloaded from The Arabidopsis Information Resource (TAIR) database (http://www.arabidopsis.org, accessed on 15 April 2020) and used as queries to screen the *B. rapa* genome database (http://brassicadb.org, accessed on 15 April 2020, version 1.5) using the BLASTP program (http://brassicadb.cn/#/BLAST/ and https://plants.ensembl.org/Multi/Tools/Blast, accessed on 15 April 2020), and genes with E value < 1 × 10^−10^ were selected. To verify that the identified genes were from the *BBX* family, all non-redundant protein sequences were checked using the following databases to ensure they were complete and included the targeted domains: NCBI-CDD (https://www.ncbi.nlm.nih.gov/Structure/cdd/wrpsb.cgi, accessed on 15 April 2020), Pfam (http://pfam.xfam.org/, accessed on 15 April 2020), and SMART (http://smart.embl-heidelberg.de/, accessed on 15 April 2020). For a comparative analysis of the *BBX* gene family in Brassicaceae, *BBX* genes were identified in two other Brassicaceae species, namely *B*. *oleracea* (http://plants.ensembl.org/index.html, accessed on 15 April 2020) and *B. napus* (http://plants.ensembl.org/index.html, accessed on 15 April 2020), using the same process. The chromosomal locations of the coding sequences and the number of encoded amino acids in the *B. rapa*, *B. oleracea*, and *B. napus* genes were determined using publicly available databases (http://brassicadb.org and http://plants.ensembl.org/index.html, accessed on 15 April 2020). The grand average of hydropathy (GRAVY) index, isoelectric point (pI), aliphatic index, molecular weight (kDa), and instability index of the BBX proteins in the three *Brassica* species were calculated using ExPASy (http://www.expasy.org/tools/, accessed on 15 April 2020). The subcellular locations of *Brassica* BBX proteins were predicted using the online tool WoLF PSORT (https://wolfpsort.hgc.jp/, accessed on 15 April 2020).

### 4.2. Chromosomal Location, Tandem Duplication, and Synteny Analyses 

The chromosomal location of all identified *Brassica BBX* genes was mapped to *B. rapa, B. oleracea,* and *B. napus* chromosomes using Mapchart software (Version 2.1) [79] based on the information available at the Brassica genome database (http://brassicadb.org, accessed on 15 April 2020). The physical location of *BBX* genes on the chromosomes was used to search the tandem duplication using the McScanX toolkit [80]. The circular map of syntenic analysis in the *Brassica* species genome was constructed using TBtools software [81].

### 4.3. Gene Structure, Motif, Conserved Domain, and Phylogenetic Analyses

On the basis of their genomic and coding sequences, the exon–intron structures of the *B. rapa*, *B. oleracea*, and *B. napus BBX* genes were analyzed using the Gene Structure Display Server. To identify the conserved domains and BBX motif, the *B. rapa*, *B. oleracea*, and *B. napus* BBX protein sequences were analyzed using the NCBI-CDD online portal (https://www.ncbi.nlm.nih.gov/Structure/cdd/wrpsb.cgi, accessed on 20 April 2020) and the MEME program (https://meme-suite.org/meme/, accessed on 15 April 2020). Multiple BBX protein sequences were aligned using ClustalW (version 2.0) and sequence logos were created using the Web logo platform (https://weblogo.berkeley.edu/logo.cgi, accessed on 15 April 2020). An unrooted neighbor-joining phylogenetic tree was constructed using MEGA 7.0 (1000 bootstrap replicates) to clarify the evolutionary relationships among 236 BBX proteins from *B. rapa*, *B. oleracea*, *B. napus*, and *A. thaliana* (51 from *B. rapa*, 52 from *B. oleracea*, 101 from *B. napus*, and 32 from *A. thaliana*).

### 4.4. Prediction of Cis-Element 

To identify the putative cis-acting elements found in *Brassica BBX* genes, the 1.5 kb genomic sequence upstream of the initiation codon (ATG) of each gene was subjected to PlantCARE (http://bioinformatics.psb.ugent.be/webtools/plantcare/html, accessed on 15 April 2020) [82].

### 4.5. Plant Materials, Growth Conditions, and Stress Treatments

*Brassica rapa* inbred line Kenshin seeds were germinated at 24 °C on moistened filter paper in Petri dishes. Four days later, healthy and uniformly growing plants were transferred to pots (16 cm diameter) and then incubated in a growth chamber at 24 °C with a 16-h light (800 μmol m^−2^ s^−1^)/8-h dark cycle and 60–65% relative humidity. Plants were watered equally daily. The effects of abiotic stress and hormone treatments on *BBX* gene expression were examined using 21-day-old seedlings. Specifically, seedlings (stem and leaf tissue) were sprayed with a solution comprising 20% polyethylene glycol 6000 (PEG6000), 200 mM mannitol, 200 mM NaCl, 50 µM abscisic acid (ABA), 100 µM gibberellic acid (GA3), 100 µM methyl jasmonate (MeJA), or 200 µM salicylic acid (SA) (pH 6.5) [83]. The mock control plants were treated with ddH_2_O. To simulate heat and cold stresses, plants were incubated in a growth chamber at 42 °C and 4 °C, respectively. Samples were collected at 0, 6, 12, and 24 h post-treatment. To simulate drought stress, watering was stopped at 12 days after germination and plants were collected 3, 6, and 9 days later. To minimize the effect of the circadian clock on gene expression, control and treated plants were collected at the same time. For all treatments, three biological replicates of plant samples were collected, immediately frozen in liquid nitrogen, and stored at −80 °C until use.

### 4.6. RNA Isolation, cDNA Synthesis, and Primer Design

Total RNA was isolated from the collected plant leaves using the RNeasy Plant Mini Kit (Qiagen, Hilden, Germany). Residual genomic DNA was eliminated via an on-column DNase treatment. The RNA quality and quantity were determined using the NanoDrop spectrophotometer (Thermo Scientific, Waltham, MA, USA) and by 1% agarose gel electrophoresis. Total RNA (1 µg) was used as the template for synthesizing cDNA using the TOPscript RT Dry mix kit (Enzynomics, Daejeon, Korea). Quantitative real-time (qRT) PCR primers were designed by IDT, Inc. (Coralville, IA, USA) and are listed in Table S17. For each primer pair, a reverse transcription PCR was performed and the amplified products were examined by 1.5% agarose gel electrophoresis to confirm they were the expected size.

### 4.7. Quantitative Real-Time PCR Analysis

The cDNA samples were diluted 5 times using nuclease-free water and then 10 ng cDNA was used for each qRT-PCR analysis. The AccuPower 2X GreenStar qPCR MasterMix (Bioneer, Daejeon, Korea) and the CFX96 Touch Real-Time PCR Detection System (Bio-Rad, Hercules, CA, USA) were used for the qRT-PCR analysis. Each 20-µL reaction comprised 5 μL SYBR mix, 1 μL cDNA, 1 μL of each primer (10 μM), and 12 μL ddH_2_O. The *Brassica ERF1* gene was used as an internal control for normalizing expression levels (Table S17). The PCR program was as follows: 94 °C for 5 min, 40 cycles of 94 °C for 15 s, 62 °C for 20 s, and 72 °C for 20 s. A melting curve analysis was performed at the end of the qRT-PCR analysis to confirm gene-specific products were amplified. The qRT-PCR was completed using three technical replicates and relative gene expression levels were calculated according to the 2^−ΔΔCt^ method [84]. The heat map showed expression patterns of 51 *BrBBX* genes in different organs and during various developmental stages using available transcriptome data [51]. The relative expression data shown in the heat map were generated using the pheatmap package of R.

## 5. Conclusions

In this study, we completed a systematic genome-wide analysis of the *BBX* gene family members in *Brassica* species. A total of 51, 52, and 101 *BBX* genes were identified in *B. rapa*, *B. oleracea*, and *B. napus*, respectively. In all three *Brassica* species, the genes in the same phylogenetic groups were very similar in terms of their conserved domains and structures. A gene duplication analysis indicated that segmental duplications were primarily responsible for the expansion of the *Brassica* BBX gene families. Several different cis-acting elements were detected in the promoter regions of the *BBX* genes in the three analyzed *Brassica* species, implying these genes are associated with complex regulatory mechanisms and networks governing developmental processes and responses to diverse abiotic stresses and exogenously applied hormones. The observed *BrBBX* expression patterns in different tissues suggest that *BrBBX*s may have multiple functions related to the development and growth of *Brassica* species. Our qRT-PCR analysis of *BrBBX* expression under various abiotic stress conditions and in response to hormones revealed that *BrBBX*s may influence abiotic stress tolerance and hormone signaling by modulating multiple stress-responsive and hormone signaling pathways. Overall, the data generated in this study may form the basis of future functional characterizations of *Brassica BBX* genes, especially regarding plant development and abiotic stress responses.

## Figures and Tables

**Table 1:** Summary of B-box genes in *B. rapa*, *B. oleracea*, *B. napus*, and *Arabidopsis thaliana*.

B-Box						
Species	Group I	Group II	Group III	Group IV	Group V	Total
*A. thaliana*	6	7	4	8	7	32
*B. rapa*	8	11	6	17	9	51
*B. oleracea*	9	11	7	16	9	52
*B. napus*	26	13	22	26	14	101

## Data Availability

All the necessary data generated are provided in the form of figures, tables, and supplementary information.

## Supplementary Materials

The following are available online at https://www.mdpi.com/article/10.3390/ijms221910367/s1. References [85,86,87,88,89,90] are cited in the Supplementary Materials.

## Abbreviations

BBX	B-box
CCT	CONSTANS, CO-like, and TOC1
*BrBBX*	Brassica rapa B-box
*BoBBX*	*BoBBX* Brassica oleracea B-box
*BnBBX*	*BnBBX* Brassica napus B-box
HMM	Hidden Markov Model
PEG	Polyethylene glycol
ABA	Abscisic acid
GA	Gibberellic acid
MeJA	Methyl jasmonate
SA	Salicylic acid
