# Supplementary material for: Genome-Wide Identification, Evolution, and Comparative Analysis of B-Box Genes in Brassica rapa, B. oleracea, and B. napus and Their Expression Profiling in B. rapa in Response to Multiple Hormones and Abiotic Stresses
Source: Int J Mol Sci. 2021 Sep 26;22(19):10367. doi: 10.3390/ijms221910367 (PMC8509055; doi:10.3390/ijms221910367)
Supplement: Supplementary file 1 [file ijms-22-10367-s001.zip › Suppl Fig. 1a_.pdf]

```
                     10        20        30        40        50        60        70        80
             ....|....|....|....|....|....|....|....|....|....|....|....|....|....|....|....|
BrBBX8       -----------------------VCNTCRSAACTVYCRADSAYLCATCDAQVHAANLL-ASRHERVRV------------ 44
BrBBX49      ----------------------QTCDTCRSAACTVYCRPDSAYLCTSCDAQIHEANRL-ASRHERVRV------------ 45
BrBBX7       ---------------------TRACDTCRSAACTIYREADSTYLCTTCDARVHAA---------KRVR------------ 38
BrBBX50      ----------------ENNTGPRACDTCGSTICTVYCHADSAYLCNSCDAQVH--------------------------- 37
BrBBX26      -----------------------LCDSCKSATAALYCRPDAAFLCLSCDSKVHAANKL-ASRHARVWM------------ 44
BrBBX42      -----------------------LCDSCKSATAALYCRPDAAFLCLSCDSKVHAANKL-ASRHARVWM------------ 44
BrBBX19      -------------------MASRPCDSCRSAAATLFCRADAAFLCGECDGKIHTANKL-ASRHERVLL------------ 48
BrBBX10      -------------------AAARSCDACKSASAAVYCRFDSAFLCITCDTSIHSFT-----RHERV-------------- 42
BrBBX29      -----------------------PCEFCGERAAVLFCRADTAKLCLPCDQHVHTANLL-SKKHVRSQI------------ 44
BrBBX51      ----------------------VPCDFCGERTAVLFCRADTAKLCLSCDHHVHKANLL-SRKHVRSRI------------ 45
BrBBX39      ----------------------VPCDFCNERAAVLFCRADAAKLCLPCDRHVHTANLL-SKKHVRSQI------------ 45
BrBBX21      ------------------------CDYCESSVALVYCKADSAKLCLACDNQVHVTNQL-FSKHFRSLL------------ 43
BrBBX6       -----------------------MCDFCGEQRSMVYCRSDAACLCLSCDRSIHSANAL-SKRHSRTLV------------ 44
BrBBX25      -----------------------MCDFCGEQRSMVYCRSDAACLCLSCDRSVHSANAL-SKRHSRTLV------------ 44
BrBBX17      -----------------------MCDFCGDQRSMVYCRSDSACLCLSCDRSVHSANAL-SKRHSRTLV------------ 44
BrBBX15      -----------------------------------YCRSDAACLCLSCDRNVHSANAL-SKRHSRTLICERCNAQPASV- 43
BrBBX27      ----------------------------------VHCRSDAACLCLSCDRNVHSANAL-SKRHSRTLI------------ 33
BrBBX5       ---------------------EPKCDYCATTQAIIYCKYDLAKLCLKCDVHVHSPNPL-SRRHMRSLI------------ 46
BrBBX48      ----------------------PMCDHCNMGKAVVYCKTHLARICSQCDRKLHHYVTMDSPDHSRLLL------------ 46
BrBBX12      ----------------------RACDSCLKRRARWYCAADDAFLCHSCDGSIHSANPL-ARRHERVLL------------ 45
BrBBX33      ----------------------RACDSCVKKRARWYCAADDAFLCHSCDSSVHSANPL-ACRHERVRL------------ 45
BrBBX30      ----------------------RACDSCVKRRARWYCAADDAFLCQSCDTLVHSANLL-ARRHERVLL------------ 45
BrBBX43      ----------------------RACDSCVKKRARWYCAADDAFLCQSCDTLVHSANPL-ARRHERVRL------------ 45
BrBBX40      ----------------------RTCDNCLKKQAHWFCAADEAFLCQSCDTSVHSANLL-ARRHERVRL------------ 45
BrBBX23      ------------------------CELCLNKHAVWYCASDDAFLCHVCDESVHRANQV-ATKHDRVCL------------ 43
BrBBX14      ------------------------CNVCEAAEAAVLCCADEAALCWACDEKIHAANKL-AEKHQRVPL------------ 43
BrBBX35      -----------------------QCNVCEAAEAAVLCCADEAALCWSCDEKVHAANKL-AGKHQRVPL------------ 44
BrBBX31      ------------------------CNVCETAEAAVLCCADEAALCLACDEKVHTANKL-AGKHQRVPL------------ 43
BrBBX28      -----------------------LCDACENAAAIVFCAADEAALCRACDEKVHMCNKL-ASRHVRV-------------- 42
BrBBX38      -----------------------LCDACENAAAIVFCAADEAALCRPCDEKVHMCNKL-ASRHVRV-------------- 42
BrBBX44      -----------------------LCDACESAAAIVFCAADEAALCCSCDEKVHMCNKL-ASRHVRV-------------- 42
BrBBX1       -----------------------WCDVCDKEEASVFCCADEAALCNGCDRHVHFANKL-AGKHLRFSL------------ 44
BrBBX37      -----------------------WCDVCDKEEASVFCCADEAALCNGCDRHVHFANKL-AGKHQRFSL------------ 44
BrBBX13      -----------------------RCDVCDKEEALVFCTADEASLCGGCDHRVH--------------------------- 30
BrBBX32      -----------------------RCDVCDKEEASVFCTADEASLCGGCDHQVHHANKL-ASKHLRFSL------------ 44
BrBBX34      -----------------------RCDVCDKEEASVFCTADEASLCDGCDHRVHHANKL-ASKHLRFSL------------ 44
BrBBX41      ------------------------CDVCEKAPATVICCADEAALCPRCDVEIHAANKL-ASKHQR--------------- 40
BrBBX46      ------------------------CDVCENAPATVICCADEAALCPKCDVEIHAANKL-ASKHQR--------------- 40
BrBBX45      ------------------------CDVCENAPATVICCADEAALCPKCDVEIHAANKL-ASKHQR--------------- 40
BrBBX20      ------------------------CDVCEKAPATLICCADEAALCASCDVEVHAANKL-ASKHQRLFL------------ 43
BrBBX22      ------------------------CDVCEKAPATLICCADEAALCAKCDVEVHAANKL-ASKHQRLFL------------ 43
BrBBX16      ----------------------------QEKAAFIFCVEDRALLCRDYDEATHA-------------------------- 26
BrBBX18      -------------------MGKKKCDLCDG-VARMYCESDQASLCWNCDGKVHGANFL-VAKHARCLL------------ 47
BrBBX36      -------------------MGKKKCDLCDG-VARMYCESDQASLCWNCDGKVHGANFL-VAKHARCLL------------ 47
BrBBX2       -----------------------KCDLCEG-VARMYCESDQASLCWNCDAKVHGANFL-VAKHTRCLL------------ 43
BrBBX11      -------------------MEKMKCELCEG-VARMFCESDQASLCWDCDGNVHGANFL-VAKHARCLL------------ 47
BrBBX47      ------------------------CELCDG-VARMFCESDQASLCWNCDGKVHGANFL-VAKHTRCLL------------ 42
BrBBX4       ---------------------PVRCELCGG-DASVFCEADTAFLCRKCDRWVH--------------------------- 31
BrBBX9       ---------------------PVRCELCGG-DASVFCEADTAFLCRKCDRWVH--------------------------- 31
BrBBX3       ----------------------VSCDLCGE-NAAVYCEADTAFLCRKCDRWVHSANF----------------------- 34
BrBBX24      ---------------------VKSCELCG-AEADLHCAADSAFLCRSCDAKFHAS------------------------- 33
BoBBX1       -----------------------WCDVCDKEEASVFCCADEAALCNGCDRHVHFANKL-AGKHLRFSL------------ 44
BoBBX19      -----------------------WCDVCDKEEASVFCCADEAALCNGCDRHVHFANKL-AGKHQRFS------------- 43
BoBBX11      -----------------------RCDVCDKEEASVFCTADEASLCGGCDHRVHHANKL-ASKHLRFSL------------ 44
BoBBX29      -----------------------RCDVCDKEEASVFCTADEASLCGGCDHQVHHANKL-ASKHLRFSL------------ 44
BoBBX32      -----------------------RCDVCDKEEASVFCTADEASLCDGCDHRVHHANKL-ASKHLRFSL------------ 44
BoBBX12      ------------------------CNVCEAAEAAVLCCADEAALCWACDEKIHAANKL-AEKHQRVPL------------ 43
BoBBX33      ------------------------CNVCEAAEAAVLCCADEAALCWACDEKVHAANKL-AGKHQRVPL------------ 43
BoBBX28      -----------------------QCNVCETAEAAVLCCADEAALCLACDEKVHAANKL-AGKHQRVPL------------ 44
BoBBX47      ----------------------IQCEVCEKAEAEVLCCSDEAALCKPCDITVHEANKL-FQRHHRVPL------------ 45
BoBBX41      -----------------------QCDVCEKAPATVICCADEAALCPKCDVEIHAANKL-ASKHQRL-------------- 42
BoBBX44      ------------------------CDVCENAPATVICCADEAALCPKCDVEIHAANKL-ASKHQR--------------- 40
BoBBX21      ------------------------CDVCEKAPATLICCADEAALCAKCDIEVHAANKL-ASKHQRLFL------------ 43
BoBBX18      -----------------------LCDACENAAAIVFCAADEAALCRPCDEKVHMCNKL-ASRHV---------------- 40
BoBBX40      -----------------------LCDACENAAAIVFCAADEAALCRACDEKVHMCNKL-ASRHVRV-------------- 42
BoBBX43      -----------------------LCDACESAAAIVFCAADEAALCCSCDEKVHMCNKL-ASRHVRV-------------- 42
BoBBX31      --------------------MERLCEVCKAYRAVVYCIADAASLCLTCDAKVHSANAL-SGRHLRTLL------------ 47
BoBBX7       ------------------------CNTCRSAACTVYCRADSAYLCATCDAQVHAANLL-ASRHERVRV------------ 43
BoBBX50      ------------------------CDTCRSAACTVYCRADSAYLCTNCDAQVHAANRL-ASRHERVRV------------ 43
BoBBX6       ----------------------RACDTCRSAACTVYREADSTYLCTNCDARVHAA---------KRVR------------ 37
```

```
BoBBX51   ----------------------PACDTCGSTICTVYCHADSAYLCNSCDAQVHSANRV-ASRHKRVRV------------ 45
BoBBX8    ----------------------------------------------------HSFT-----RHERV-------------- 9
BoBBX49   ----------------------RFCDACQSVSAAVFCRVDSAFLCLTCDTRIHSYT-----RHERVFL------------ 41
BoBBX38   -----------------------LCDSCKSATAALYCRPDAAFLCLSCDSKVHAANKL-ASRHARVWM------------ 44
BoBBX46   -----------------------LCDSCKSATAALYCRPDAAFLCLSCDSKVHAANKL-ASRHARVWM------------ 44
BoBBX22   -------------------MASRPCDSCRSAAATLLCRADAAFLCGECDGKIHSANKL-ASRHERVWL------------ 48
BoBBX10   ----------------------RACDSCLKRRARWFCAADDAFLCHSCDGSVHSANPL-ARRHERVLL------------ 45
BoBBX30   ----------------------RACDNCVKKRARWYCAADDAFLCHFCDGSVHSANPL-ACRHERVRL------------ 45
BoBBX35   ----------------------RACDSCVKRRARWYCAADDAFLCQSCDTLVHSANLL-ARRHERVLL------------ 45
BoBBX36   ----------------------RACDSCVKRRARWYCAADDAFLCQSCDTLVHSANLL-ARRHERVLL------------ 45
BoBBX23   ----------------------RACDSCVKKRARWYCAADDAFLCQSCDTLVHSANPL-ARRHERVRL------------ 45
BoBBX16   ----------------------RTCDNCFKKRAHWFCAADEAFLCQSCDTSVHSANLL-ARRHERVRL------------ 45
BoBBX24   ------------PERQEDVKRPRDCELCLNKHAVWYCASDDAFLCHVCDESVHRANQV-ATKHDRVCL------------ 55
BoBBX4    ---------------------EPKCDYCATTQAIIYCKYDLAKLCLKCDVHVHSPNPL-SRRHMRSLI------------ 46
BoBBX45   ----------------------PMCDHCNMGKAVVYCKTHLARICSQCDRKLHHYVTMDSPDHSRLLL------------ 46
BoBBX5    -----------------------MCDFCGEQRSMVYCRSDDACLCLSCDRSIHSANAL-SKRHSRTLV------------ 44
BoBBX27   -----------------------MCDFCGEQRSMVYCRSDAACLCLSCDRSVHSANAL-SKRHSRTLV------------ 44
BoBBX14   -----------------------MCDFCGEQRSMVYCRSDSACLCLSCDRSVHSANAL-SKRHSRTLV------------ 44
BoBBX13   -----------------------ICDFCNEQRSMVYCRSDAACLCLSCDRNVHSANAL-SKRHSRTLI------------ 44
BoBBX17   --------------------ERVPCDFCNERAAVLFCRADAAKLCLPCDRHVHTANLL-SKKHVRSQI------------ 47
BoBBX34   -----------------------PCDFCGERAAVLFCRADTAKLCLPCDQQVHTANLL-SKKHVRSQI------------ 44
BoBBX52   -----------------------PCDFCGERTAFLFCRADAAKLCLPCDHRVHKANLL-SRKHVRSRI------------ 44
BoBBX20   ------------------------CDYCESSVALVYCKADSAKLCLACDNQVHVTNQL-FSKHFRSLL------------ 43
BoBBX2    -----------------------KCDLCDG-VARMYCESDQASLCWNCDAKVHGANFL-VAKHTRCLL------------ 43
BoBBX39   -----------------------KCDLCEG-VARMYCESDQASLCWDCDGKVHGANFL-VAKHTRCLL------------ 43
BoBBX42   -------------------MGKKKCALCDG-VARMYCESDQASLCWNCDGKVHGANFL-VAKHTRCLL------------ 47
BoBBX9    -------------------MEKMKCELCEG-VARMFCESDQASLCWDCDGNVHGANFL-VAKHARCLL------------ 47
BoBBX48   -------------------MAKKKCELCDR-VARTFCESDQASLCWDCEGKVHGANFL-VAKHTRCLL------------ 47
BoBBX3    ---------------------PVRCELCGG-DASVFCEADTAFLCRKCDRWVH--------------------------- 31
BoBBX25   --------------------------------ASVFCEADSAFLCRKCDRWVH--------------------------- 21
BoBBX15   -----------------------LCELCG-AEAHLHCDADSAYLCRSCDVKFHASNFL-FSRHVRR-------------- 41
BoBBX26   ---------------------VKSCELCG-AEADLHCAADSAFLCRSCDAKFHASNIL-FSRHVRRTI------------ 45
BnABBX41  -------------------MGKKNCELCDG-VARMFCESDQASLCWNCDGKVHGANFL-VAKHTRCLL------------ 47
BnCBBX90  -------------------MAKKKCELCDR-VARMFCESDQASLCWDCDGKVHGANFL-VAKHTRCLL------------ 47
BnABBX7   -------------------MEKMKCELCEG-VARMFCESDQASLCWDCDGNVHGANFL-VAKHARCLL------------ 47
BnABBX2   ----------------------VSCDLCGE-NAAVYCEADTAFLCRKCDRWVHSA------------------------- 32
BnCBBX53  ----------------------VSCDLCGE-NAAVYCEADTAFLCRKCDRWVHSA------------------------- 32
BnABBX5   -------------------SVPVRCELCGG-DASVFCEADTAFLCRKCDRWVHGANFL-AWRHVRRVLCTACQKLTRKCL 59
BnCBBX66  ---------------------PVRCELCGG-DASVFCEADTAFLCRKCDRWVH--------------------------- 31
BnCBBX99  NEEENRSVHGGCRSLCTRPSVPVRCELCGG-DASVFCEADTAF------------------------------------- 42
BnCBBX95  --------------------------------ASVFCEADSAFLCRKCDRWVH--------------------------- 21
BnABBX20  ---------------------VKSCELCG-AEADLHCAADSAFLCRSCDAKFHAS------------------------- 33
BnCBBX70  ---------------------VKSCELCG-AEADLHCAADSAFLCRSCDAKFHASNIL-FSRHVRRTIC----------- 46
BnCBBX61  -----------------------LCELCG-AEAHLHCDADSAYLCRSCDVKFHASNFL-FSRHVRRAI------------ 43
BnABBX29  ----------------------RLCEVCKAYRAVVYCIADAASLCLTCDAKVHSANAL-SGRHLRTLLCGFCKNQP---- 53
BnCBBX76  ----------------------RLCEVCKAYRAVVYCIADAASLCLTCDAKVHSANAL-SGRHLRTLLCGFCKNQP---- 53
BnABBX1   -----------------------WCDVCDKEEASVFCCADEAALCNGCDRHVH--------------------------- 30
BnCBBX51  -----------------------WCDVCDKEEASVFCCADEAALCNGCDRHVHFANKL-AGKHLRFSLT----------- 45
BnABBX32  -----------------------WCDVCDKEEASVFCCADEAALCNGCDRHVHFANKL-AGKHQRFSLT----------- 45
BnCBBX64  -----------------------WCDVCDKEEASVFCCADEAALCNGCDRHVHFANKL-AGKHQRFSLT----------- 45
BnABBX9   -----------------------RCDVCDKEEALVFCTADEASLCGGCDHRVH--------------------------- 30
BnCBBX57  -----------------------RCDVCDKEEASVFCTADEASLCGGCDHRVHHANKL-ASKHLRFSL------------ 44
BnABBX27  -----------------------RCDVCDKEEASVFCTADEASLCGGCDHQVHHANKL-ASKHLRFSL------------ 44
BnCBBX74  -----------------------RCDVCDKEEASVFCTADEASLCGGCDHQVHHANKL-ASKHLRFSL------------ 44
BnABBX44  -----------------------RCDVCDKEEASVFCTADEASLCDGCDHRVHHANKL-ASKHLRFSL------------ 44
BnCBBX78  -----------------------RCDVCDKEEASVFCTADEASLCDGCDHRVHHANKL-ASKHLRFSL------------ 44
BnABBX10  ------------------------CNVCEAAEAAVLCCADEAALCWACDEKIHAANKL-AEKHQRVPL------------ 43
BnCBBX58  ------------------------CNVCEAAEAAVLCCADEAALCWACDEKIHAANKL-AEKHQRVPL------------ 43
BnCBBX77  ------------------------CNVCEAAEAAVLCCADEAALCWSCDEKVHAANKL-AGKHQRVPL------------ 43
BnCBBX79  ------------------------CNVCEAAEAAVLCCADEAALCWACDEKVHAANKL-AGKHQRVPL------------ 43
BnABBX30  ------------------------CNVCEAAEAAVLCCADEAALCWSCDEKVHAANKL-AGKHQRVPL------------ 43
BnABBX48  ------------------------CNVCETAEAAVLCCADEAALCLACDEKVHTANKL-AGKHQRVPL------------ 43
BnCBBX73  ------------------------CNVCETAEAAVLCCADEAALCLACDEKVHAANKL-AGKHQRVPL------------ 43
BnABBX38  -----------------------QCEVCEKAEAEVLCCSDEAALCKPCDITVHEANKL-FQRHHRVPL------------ 44
BnCBBX89  ----------------------IQCEVCEKAEAEVLCCSDEAALCKPCDITVHEANKL-FQRHHRVPL------------ 45
BnABBX16  ------------------------CDVCEKAPATLICCADEAALCASCDVEVHAANKL-ASKHQRLFL------------ 43
BnCBBX68  ------------------------CDVCEKAPATLICCSDEAALCASCDVEVHAANKL-ASKHQRLFL------------ 43
BnABBX19  ------------------------CDVCEKAPATLICCADEAALCAKCDIEVHAANKL-ASKHQRLFL------------ 43
BnCBBX98  ------------------------CDVCEKAPATLICCADEAALCAKCDIEVHAANKL-ASKHQRLFL------------ 43
BnABBX13  ----------------------------QEKAAFIFCVEDRALLCRDYDEATHA-------------------------- 26
BnABBX36  ------------------------CDVCEKAPATVICCADEAALCPRCDVEIHAANKL-ASKHQR--------------- 40
BnCBBX84  ------------------------CDVCEKAPATVICCADEAALCPKCDVEIHAANKL-ASKHQR--------------- 40
```

```
BnABBX40    ------------------------CDVCENAPATVICCADEAALCPKCDVEIHAANKL-ASKHQR--------------- 40
BnCBBX69    ------------------------CDVCENAPATVICCADEAALCPKCDVEIHAANKL-ASKHQR--------------- 40
BnABBX39    ------------------------CDVCENAPATVICCADEAALCPKCDVEIHAANKL-ASKHQR--------------- 40
BnCBBX87    ------------------------CDVCENAPATVICCADEAALCPKCDVEIHAANKL-ASKHQR--------------- 40
BnABBX33    -----------------------LCDACENAAAIVFCAADEAALCRPCDEKVHMCNKL-ASRHVR--------------- 41
BnCBBX63    -----------------------LCDACENAAAIVFCAADEAALCRPCDEKVHMCNKL-ASRHVR--------------- 41
BnCBBX83    -----------------------LCDACENAAAIVFCAADEAALCRACDEKVHMCNKL-ASRHVR--------------- 41
BnABBX25    ---------------------APCCDICENAPAFFYCEIDGSSLCLQCDMVVHVGGK---RTHGRFLL------------ 44
BnCBBX85    -----------------------LCDVCESAAAIVFCAADEAALCCSCDEKVHMCNKL-ASRHVR--------------- 41
BnABBX3     ------------------------CESCLKADADSFCPQHNKFLCYNCDIKIH--------------------------- 29
BnABBX8     ----------------------RACDSCLKRRARWYCAADDAFLCHSCDGSVHSANPL-ARRHERVLL------------ 45
BnCBBX56    ---------------------ARACDSCLKRRARWFCAADDAFLCHSCDGSVHSANPL-ARRHERVL------------- 45
BnCBBX75    ----------------------RACDNCVKKRARWYCAADDAFLCHFCD------------------------------- 27
BnABBX28    ----------------------RACDSCVKKRARWYCAADDAFLCHSCD------------------------------- 27
BnABBX31    ---------------------ARACDSCVKRRARWYCAADDAFLCQSCDTLVHSANLL-ARRHERVLL------------ 46
BnCBBX97    ---------------------ARACDSCVKRRARWYCAADDAFLCQSCDTLVHSANLL-ARRHERVLL------------ 46
BnCBBX80    -----------------------ACDSCVKRRARWYCAADDAFLCQSCDTLVHSANLL-ARRHERVLL------------ 44
BnABBX46    -----------------------ACDSCVKKRARWYCAADDAFLCQSCDTLVHSANPL-ARRHERVRL------------ 44
BnCBBX72    -----------------------ACDSCVKKRARWYCAADDAFLCQSCDTLVHSANPL-ARRHERVRLK----------- 45
BnABBX35    ----------------------RTCDNCLKKQAHWFCAADEAFLCQSCDTSVHSANLL-ARRHERVRL------------ 45
BnCBBX94    ----------------------RTCDNCLKKRAHWFCAADEAFLCQSCDTSVHSANLL-ARRHERVPL------------ 45
BnABBX22    ------------------------CELCLNKHAVWYCASDDAFLCHVCDESVHRANQV-ATKHDRVCL------------ 43
BnABBX6     -----------------------VCNTCRSAACTVYCRADSAYLCSTCDAQVHAANLL-ASRHERVRV------------ 44
BnCBBX54    -----------------------ACNTCRSAACTVYCRADSAYLCATCDAQVHAANLL-ASRHERVRV------------ 44
BnABBX42    ------------------------CDTCRSAACTVYCRADSAYLCTSCDAQIHAANRL-ASRHERVRV------------ 43
BnCBBX92    ------------------------CDTCRSAACTVYCRADSAYLCTNCDAQVHAANRL-ASRHERVRV------------ 43
BnABBX43    ----------------------RACDTCGSTICTVYCHADSAYLCNSCDAQVHSANRV-ASRHKRVRV------------ 45
BnCBBX93    ----------------------RACDTCGSTICTVYCHADSAYLCNSCDAQVHSANRV-ASRHKRVRV------------ 45
BnABBX49    ----------------------RACGTCRSAACTIYREADSTYLCTNCDARVHAA--------KRVR------------- 37
BnABBX50    ----------------------RACDTCRSAACTIYREADSTYLCTTCDARVHAA--------KRVR------------- 37
BnABBX12    ----------------------RSCDACKSASAAVYCRFDSAFLCITCDTSIHSFT-----RHERV-------------- 39
BnCBBX55    ----------------------RSCDACKSASAAVFCRVDSAFLCITCDASIHSFT-----RHERV-------------- 39
BnCBBX91    ----------------------RSCDACQSVSAAVFCRVDSAFLCLTCDTRIHSYT-----RHERVFL------------ 41
BnABBX15    -------------------MASRPCDSCRSAAATLFCRADAAFLCGECDGKIHTANKL-ASRHERVLL------------ 48
BnCBBX67    -------------------MASRPCDSCRSAAATLFCRADAAFLCGECDGKIHSANKL-ASRHERVWL------------ 48
BnABBX23    -----------------------LCDSCKSATAALYCRPDAAFLCLSCDSKVHAANKL-ASRHARVWM------------ 44
BnCBBX82    -------------------MASKLCDSCKSATAALYCRPDAAFLCLSCDSKVHAANKL-ASRHARVWM------------ 48
BnABBX37    -----------------------LCDSCKSATAALYCRPDAAFLCLSCDSKVHAANKL-ASRHARVWM------------ 44
BnCBBX88    -----------------------LCDSCKSATAALYCRPDAAFLCLSCDSKVHAANKL-ASRHARVWM------------ 44
BnABBX45    ----------------------PMCDHCNMGKAVVYCKTHLARICSQCDRKLHHYVTMDSPDHSRLLL------------ 46
BnCBBX86    ----------------------PMCDHCNMGKAVVYCKTHLARICSQCDRKLHHYVTMDSPDHSRLLL------------ 46
BnABBX4     ---------------------EPKCDYCATTQAIIYCKYDLAKLCLKCDVHVHSPNPL-SRRHMRSLI------------ 46
BnCBBX100   ---------------------EPKCDYCATTQAIIYCKYDLAKLCLKCDVHVHSPNPL-SRRHMRSLI------------ 46
BnABBX14    -----------------------MCDFCGDQRSMVYCRSDSACLCLSCDRSVHSANAL-SKRHSRTLV------------ 44
BnCBBX60    -----------------------MCDFCGEQRSMVYCRSDSACLCLSCDRSVHSANAL-SKRHSRTLV------------ 44
BnABBX21    -----------------------MCDFCGEQRSMVYCRSDAACLCLSCDRSVHSANAL-SKRHSRTLV------------ 44
BnCBBX71    -----------------------MCDFCGEQRSMVYCRSDAACLCLSCDRSVHSANAL-SKRHSRTLV------------ 44
BnCBBX52    -----------------------MCDFCGEQRSMVYCRSDAACLCLSCDRSIHSANAL-SKRHSRTLV------------ 44
BnABBX11    -----------------------MCDFCNEQRSMVYCRSDAACLCLSCDRNVHSANAL-SKRHSRTLI------------ 44
BnCBBX59    -----------------------ICDFCNEQRSMVYCRSDAACLCLSCDRNVHSANAL-SKRHSRTLI------------ 44
BnABBX24    -----------------------MCDFCGEQRSMVHCRSDAACLCLSCDRNVHSANAL-SKRHSRTLI------------ 44
BnCBBX81    -----------------------MCDFCGEQRSMVHCRSDAACLCLSCDRNVHSANAL-SKRHSRTLI------------ 44
BnCBBX96    ------------------------CDFCGERTAVLFCRADAAKLCLPCD------------------------------- 25
BnABBX17    --------------------ERVPCDFCGERTAVLFCRADAAKLCLSCDHHVHKANLL-SRKHVRSLI------------ 47
BnABBX47    --------------------ERVPCDFCGERTAVLFCRADAAKLCLSCDHHVHKANLL-SRKHVRSLI------------ 47
BnABBX26    ------------------------CEFCGERAAVLFCRADTAKLCLPCDQHVHTANLL-SKKHVRSQI------------ 43
BnCBBX101   -----------------------PCDFCGERTAVLFCRADTAKLCLPCDQQVHTANLL-SKKHVRSQI------------ 44
BnABBX34    ----------------------VPCDFCNERAAVLFCRADAAKLCLPCDRHVHTANLL-SKKHVRSQI------------ 45
BnCBBX62    --------------------ERVPCDFCNERAAVLFCRADAAKLCLPCDRHVHTANLL-SKKHVRSQI------------ 47
BnABBX18    ------------------------CDYCESSVALVYCKADSAKLCLACDNQVHVTNQL-FSKHFRSLL------------ 43
BnCBBX65    ------------------------CDYCESSVALVYCKADSAKLCLACDNQVHVTNQL-FSKHFRSLL------------ 43

                    90        100       110
            ....|....|....|....|....|....|....|..
BrBBX8      ------------------------------------- 44
BrBBX49     ------------------------------------- 45
BrBBX7      ------------------------------------- 38
BrBBX50     ------------------------------------- 37
BrBBX26     ------------------------------------- 44
BrBBX42     ------------------------------------- 44
BrBBX19     ------------------------------------- 48
BrBBX10     ------------------------------------- 42
```

```
BrBBX29    ------------------------------------- 44
BrBBX51    ------------------------------------- 45
BrBBX39    ------------------------------------- 45
BrBBX21    ------------------------------------- 43
BrBBX6     ------------------------------------- 44
BrBBX25    ------------------------------------- 44
BrBBX17    ------------------------------------- 44
BrBBX15    ------------------------------------- 43
BrBBX27    ------------------------------------- 33
BrBBX5     ------------------------------------- 46
BrBBX48    ------------------------------------- 46
BrBBX12    ------------------------------------- 45
BrBBX33    ------------------------------------- 45
BrBBX30    ------------------------------------- 45
BrBBX43    ------------------------------------- 45
BrBBX40    ------------------------------------- 45
BrBBX23    ------------------------------------- 43
BrBBX14    ------------------------------------- 43
BrBBX35    ------------------------------------- 44
BrBBX31    ------------------------------------- 43
BrBBX28    ------------------------------------- 42
BrBBX38    ------------------------------------- 42
BrBBX44    ------------------------------------- 42
BrBBX1     ------------------------------------- 44
BrBBX37    ------------------------------------- 44
BrBBX13    ------------------------------------- 30
BrBBX32    ------------------------------------- 44
BrBBX34    ------------------------------------- 44
BrBBX41    ------------------------------------- 40
BrBBX46    ------------------------------------- 40
BrBBX45    ------------------------------------- 40
BrBBX20    ------------------------------------- 43
BrBBX22    ------------------------------------- 43
BrBBX16    ------------------------------------- 26
BrBBX18    ------------------------------------- 47
BrBBX36    ------------------------------------- 47
BrBBX2     ------------------------------------- 43
BrBBX11    ------------------------------------- 47
BrBBX47    ------------------------------------- 42
BrBBX4     ------------------------------------- 31
BrBBX9     ------------------------------------- 31
BrBBX3     ------------------------------------- 34
BrBBX24    ------------------------------------- 33
BoBBX1     ------------------------------------- 44
BoBBX19    ------------------------------------- 43
BoBBX11    ------------------------------------- 44
BoBBX29    ------------------------------------- 44
BoBBX32    ------------------------------------- 44
BoBBX12    ------------------------------------- 43
BoBBX33    ------------------------------------- 43
BoBBX28    ------------------------------------- 44
BoBBX47    ------------------------------------- 45
BoBBX41    ------------------------------------- 42
BoBBX44    ------------------------------------- 40
BoBBX21    ------------------------------------- 43
BoBBX18    ------------------------------------- 40
BoBBX40    ------------------------------------- 42
BoBBX43    ------------------------------------- 42
BoBBX31    ------------------------------------- 47
BoBBX7     ------------------------------------- 43
BoBBX50    ------------------------------------- 43
BoBBX6     ------------------------------------- 37
BoBBX51    ------------------------------------- 45
BoBBX8     ------------------------------------- 9
BoBBX49    ------------------------------------- 41
BoBBX38    ------------------------------------- 44
BoBBX46    ------------------------------------- 44
BoBBX22    ------------------------------------- 48
BoBBX10    ------------------------------------- 45
BoBBX30    ------------------------------------- 45
BoBBX35    ------------------------------------- 45
BoBBX36    ------------------------------------- 45
```

```
BoBBX23    ------------------------------------- 45
BoBBX16    ------------------------------------- 45
BoBBX24    ------------------------------------- 55
BoBBX4     ------------------------------------- 46
BoBBX45    ------------------------------------- 46
BoBBX5     ------------------------------------- 44
BoBBX27    ------------------------------------- 44
BoBBX14    ------------------------------------- 44
BoBBX13    ------------------------------------- 44
BoBBX17    ------------------------------------- 47
BoBBX34    ------------------------------------- 44
BoBBX52    ------------------------------------- 44
BoBBX20    ------------------------------------- 43
BoBBX2     ------------------------------------- 43
BoBBX39    ------------------------------------- 43
BoBBX42    ------------------------------------- 47
BoBBX9     ------------------------------------- 47
BoBBX48    ------------------------------------- 47
BoBBX3     ------------------------------------- 31
BoBBX25    ------------------------------------- 21
BoBBX15    ------------------------------------- 41
BoBBX26    ------------------------------------- 45
BnABBX41   ------------------------------------- 47
BnCBBX90   ------------------------------------- 47
BnABBX7    ------------------------------------- 47
BnABBX2    ------------------------------------- 32
BnCBBX53   ------------------------------------- 32
BnABBX5    VGDNYHVVLPSATAGEAAVEDITIRSEQDSNDDEVPY 96
BnCBBX66   ------------------------------------- 31
BnCBBX99   ------------------------------------- 42
BnCBBX95   ------------------------------------- 21
BnABBX20   ------------------------------------- 33
BnCBBX70   ------------------------------------- 46
BnCBBX61   ------------------------------------- 43
BnABBX29   ------------------------------------- 53
BnCBBX76   ------------------------------------- 53
BnABBX1    ------------------------------------- 30
BnCBBX51   ------------------------------------- 45
BnABBX32   ------------------------------------- 45
BnCBBX64   ------------------------------------- 45
BnABBX9    ------------------------------------- 30
BnCBBX57   ------------------------------------- 44
BnABBX27   ------------------------------------- 44
BnCBBX74   ------------------------------------- 44
BnABBX44   ------------------------------------- 44
BnCBBX78   ------------------------------------- 44
BnABBX10   ------------------------------------- 43
BnCBBX58   ------------------------------------- 43
BnCBBX77   ------------------------------------- 43
BnCBBX79   ------------------------------------- 43
BnABBX30   ------------------------------------- 43
BnABBX48   ------------------------------------- 43
BnCBBX73   ------------------------------------- 43
BnABBX38   ------------------------------------- 44
BnCBBX89   ------------------------------------- 45
BnABBX16   ------------------------------------- 43
BnCBBX68   ------------------------------------- 43
BnABBX19   ------------------------------------- 43
BnCBBX98   ------------------------------------- 43
BnABBX13   ------------------------------------- 26
BnABBX36   ------------------------------------- 40
BnCBBX84   ------------------------------------- 40
BnABBX40   ------------------------------------- 40
BnCBBX69   ------------------------------------- 40
BnABBX39   ------------------------------------- 40
BnCBBX87   ------------------------------------- 40
BnABBX33   ------------------------------------- 41
BnCBBX63   ------------------------------------- 41
BnCBBX83   ------------------------------------- 41
BnABBX25   ------------------------------------- 44
BnCBBX85   ------------------------------------- 41
BnABBX3    ------------------------------------- 29
```

```
BnABBX8    ------------------------------------- 45
BnCBBX56   ------------------------------------- 45
BnCBBX75   ------------------------------------- 27
BnABBX28   ------------------------------------- 27
BnABBX31   ------------------------------------- 46
BnCBBX97   ------------------------------------- 46
BnCBBX80   ------------------------------------- 44
BnABBX46   ------------------------------------- 44
BnCBBX72   ------------------------------------- 45
BnABBX35   ------------------------------------- 45
BnCBBX94   ------------------------------------- 45
BnABBX22   ------------------------------------- 43
BnABBX6    ------------------------------------- 44
BnCBBX54   ------------------------------------- 44
BnABBX42   ------------------------------------- 43
BnCBBX92   ------------------------------------- 43
BnABBX43   ------------------------------------- 45
BnCBBX93   ------------------------------------- 45
BnABBX49   ------------------------------------- 37
BnABBX50   ------------------------------------- 37
BnABBX12   ------------------------------------- 39
BnCBBX55   ------------------------------------- 39
BnCBBX91   ------------------------------------- 41
BnABBX15   ------------------------------------- 48
BnCBBX67   ------------------------------------- 48
BnABBX23   ------------------------------------- 44
BnCBBX82   ------------------------------------- 48
BnABBX37   ------------------------------------- 44
BnCBBX88   ------------------------------------- 44
BnABBX45   ------------------------------------- 46
BnCBBX86   ------------------------------------- 46
BnABBX4    ------------------------------------- 46
BnCBBX100  ------------------------------------- 46
BnABBX14   ------------------------------------- 44
BnCBBX60   ------------------------------------- 44
BnABBX21   ------------------------------------- 44
BnCBBX71   ------------------------------------- 44
BnCBBX52   ------------------------------------- 44
BnABBX11   ------------------------------------- 44
BnCBBX59   ------------------------------------- 44
BnABBX24   ------------------------------------- 44
BnCBBX81   ------------------------------------- 44
BnCBBX96   ------------------------------------- 25
BnABBX17   ------------------------------------- 47
BnABBX47   ------------------------------------- 47
BnABBX26   ------------------------------------- 43
BnCBBX101  ------------------------------------- 44
BnABBX34   ------------------------------------- 45
BnCBBX62   ------------------------------------- 47
BnABBX18   ------------------------------------- 43
BnCBBX65   ------------------------------------- 43
```
