# Supplementary material for: Genome-Wide Identification, Evolution, and Comparative Analysis of B-Box Genes in Brassica rapa, B. oleracea, and B. napus and Their Expression Profiling in B. rapa in Response to Multiple Hormones and Abiotic Stresses
Source: Int J Mol Sci. 2021 Sep 26;22(19):10367. doi: 10.3390/ijms221910367 (PMC8509055; doi:10.3390/ijms221910367)
Supplement: Supplementary file 1 [file ijms-22-10367-s001.zip › Suppl Fig. 1b_.pdf]

```
                   10        20        30        40        50        60        70
          ....|....|....|....|....|....|....|....|....|....|....|....|....|....|.
BoBBX1    ------PLCDICGERRALLFCQKDRAILCRECDIPIHQANEHTKKHNRFLL-------------------- 45
BoBBX19   ------PLCDICGERRALLFCQEDRAILCRECDIPIHQANEHTKKHNRFLL-------------------- 45
BoBBX11   ------PICDICQEKKALLFCQQDRAILCKDCDSSIHSANEQVKKHDRFLL-------------------- 45
BoBBX29   -------ICDICQEKKALLFCQQDRAILCKDCDTSIHSANEHKKKHDRFLL-------------------- 44
BoBBX32   --------CDICQEKKALLFCQQDRAILCKDCDSSIHSANEHTKKHDRFLL-------------------- 43
BoBBX12   ------PKCDICQEASGFFFCLQDRALLCRKCDVAIHTVNPHVSAHQRFLL-------------------- 45
BoBBX33   ------PKCDICQEASGFFFCLQDRALLCRKCDVAIHTVNPHVSAHQRFLL-------------------- 45
BoBBX28   ----SKPKCDICQEATGFFFCLQDRALLCRKCDVSIHTVNPHVSAHQRFLL-------------------- 47
BoBBX47   TTSSAAPLCDICKERKGYFFCLDDRALLCNDCDGAIHIC----SSHQRFLL-------------------- 47
BoBBX41   ------RRCDICQEKAAFIFCVEDRALLCRDCDESIHVANSRSANHQRFLA-------------------- 45
BoBBX44   ------PRCDICQEKAAFIFCVEDRALLCKDCDESIHLANSRSANHQRLL--------------------- 44
BoBBX21   ------PPCDICLEKAAFIFCVEDRALLCRDCDEGTHAPNTRSANHQRFL--------------------- 44
BoBBX18   -----APCCDICENAPAFFYCEIDGSSLCLQCDMVVHVGGKR--THGRFLL-------------------- 44
BoBBX40   -----APCCDICENAPAFFYCEIDGSSLCLQCDMVVHVGGKR--THGRFLL-------------------- 44
BoBBX43   -----APSCDICENAPAFFYCEIDGTSLCLQCDMVVHVGGKR--THRRFLL-------------------- 44
BoBBX31   --------CGFCKNQPCVVRCLDHKMFLCNGCNEKIHGVVS--SKHVRHDV-------------------- 41
BoBBX7    ---ERVRVCEACERAPAAFFCKADAASLCIACDSQVHLANPLARRHQRVPI-------------------- 48
BoBBX50   ---ERVRVCQSCERAPAAFFCKADAASLCTACDSQIHSANPLARRHQRVPI-------------------- 48
BoBBX6    --AKRVRVGDSCESAPAAFLCKADAASLCTACDAEIHSANPLASRHQRVPISANS---------------- 53
BoBBX51   ---KRVRVCESCERAPAAFMCEADDVSLCTACDLEVHSANPLARRHQRVPV-------------------- 48
BoBBX8    -------ICEVCEQAPAAVTCKADAASLCVTCDSDIHSANPLASRHERVPV-------------------- 44
BoBBX49   -------LCEVCEQAPAAVTCKADAASLCVTCDSDIHSANPLASRHERVPV-------------------- 44
BoBBX38   ----RVWMCEVCEQAPAHVTCKADAAALCVTCDRDIHSANPLARRHARR---------------------- 45
BoBBX46   ----RVWMCEVCEQAPAHVTCKADAAALCVTCDRDIHSANPLARRHERVPV-------------------- 47
BoBBX22   ----RVWLCQVCEQSPAHVTCKADAAALCVTCDRDIHSANPLSRRHERVPV-------------------- 47
BoBBX5    -------VCERCNAQPATVRCVEERVSLCQNCDW------------------------------------- 27
BoBBX27   -------VCERCNAQPATVRCVEERVSLCQNCDWSGH---------------------------------- 30
BoBBX14   -------VCERCNAQPATVRCVEERVSLCQNCDWSGH---------------------------------- 30
BoBBX13   -------ICERCNAQPASVRCSDERVSLCQNCDWSGHNND------------------------------- 33
BoBBX37   --------CERCNAQPASVRCTDERVSLCQNCDWLGH---------------------------------- 29
BoBBX17   -------ICDNCGSEPVSVRCFTDSLVLCQECDWDVH---------------------------------- 30
BoBBX34   -------ICDNCGNEPVSVRCFTDNLVLCQDCDWDVH---------------------------------- 30
BoBBX52   ------RICDNCGSEPVSVRCFTDDLVLCLECDWDVH---------------------------------- 31
BoBBX20   -------LCDSCDDSPSSVFCDTESSVLCQNCDWQ------------------------------------ 28
BnABBX1   ------PLCDICGERRALLFCQEDRAILCRECDIPIHQANEHTKKHNRFLL-------------------- 45
BnCBBX51  ------PLCDICGERRALLFCQEDRAILCRECDIPIHQANEHTKKHNRFLL-------------------- 45
BnABBX32  ---KDAPLCDICGKRRALLFCQEDRAILCRECDIPIHQANEHTKKHNRFLLTGVKISASPSTYPKASNSIS 68
BnCBBX64  ------PLCDICGERRALLFCQEDRAILCRECDIPIHQANEHTKKHNRFLLTGVKISASPSTYPKASNSNS 65
BnABBX9   -------ICDICQEKKALLFCQEDRAILCKDCDSSIHSANEHVKKHDRFLL-------------------- 44
BnCBBX57  ------PICDICQEKKALLFCQQDRAILCKDCDSSIHSANEQVKKHDRFLL-------------------- 45
BnABBX27  -------ICDICQEKKALLFCQQDRAILCKDCDTSIHSANEHKKKHDRFLL-------------------- 44
BnABBX44  ---NSSPICDICQEKKALLFCQQDRAILCKDCDSSIHSANEHTKKHDRFLL-------------------- 48
BnCBBX78  --------CDICQEKKALLFCQQDRAILCKDCDSSIHSANEHTKKHDRFLL-------------------- 43
BnABBX10  ------PKCDICQEASGFFFCLQDRALLCRKCDVAIHTVNPHVSAHQRFLL-------------------- 45
BnCBBX58  ------PKCDICQEASGFFFCLQDRALLCRKCDVAIHTVNPHVSAHQRFLL-------------------- 45
BnCBBX77  ------------QEASGFFFCLQDRALLCRKCDVAIHTVNPRVSAHQRFLL-------------------- 39
BnCBBX79  ------PKCDICQEASGFFFCLQDRALLCRKCDVAIHTVNPRVSAHQRFLL-------------------- 45
BnABBX30  ------PKCDICQEASGFFFCLQDRALLCRKCDVAIHTVNPHVSAHQRFLL-------------------- 45
BnABBX48  ------PKCDICQEATGFFFCLQDRALLCRKCDVAIHTVNPHVSAHQRFLL-------------------- 45
BnCBBX73  ----SKPKCDICQEATGFFFCLQDRALLCRKCDVSIHTVNPHVSAHQRFLL-------------------- 47
BnABBX38  ---STAPLCDICKERKGYFFCLDDRALLCNDCDGAIHIC----NSHQRFLL-------------------- 44
BnCBBX89  ----AAPLCDICKERKGYFFCLDDRALLCNDCDGAIHIC----SSHQRFLL-------------------- 43
BnABBX16  ------PPCDICLEKAAFIFCVEDRALLCRDCDEATHAPNTRSANHQRFL--------------------- 44
BnCBBX68  ------PPCDICLEKAAFIFCVEDRALLCRDCDEATHAPNTRSANHQRFL--------------------- 44
BnABBX19  ------PPCDICLEKAAFIFCVEDRALLCRDCDEGTHAPNTRSANHQRFL--------------------- 44
BnCBBX98  ------PPCDICLEKAAFIFCVEDRALLCRDCDEGTHAPNTRSANHQRFL--------------------- 44
BnABBX36  ------PRCDICQEKAAFIFCVEDRALLCRDCDESIHVANTRSANHQRLL--------------------- 44
BnCBBX84  ------PRCDICQEKAAFIFCVEDRALLCRDCDESIHVANTRSANHQRFL--------------------- 44
BnABBX40  ------PRCDICQEKAAFIFCVEDRALLCRDCDESIHVANSRSANHQRFL--------------------- 44
BnCBBX69  ------PRCDICQEKAAFIFCVEDRALLCRDCDESIHVANSRSANHQRFL--------------------- 44
BnABBX39  ------PRCDICQEKAAFIFCVEDRALLCKDCDESIHVANSRSANHQRLL--------------------- 44
BnCBBX87  ------PRCDICQEKAAFIFCVEDRALLCKDCDESIHLANSRSANHQRLL--------------------- 44
BnABBX33  -----APCCDICENAPAFFYCEIDGSSLCLQCDMVVHVGGKR--THGRFLL-------------------- 44
BnCBBX63  -----APCCDICENAPAFFYCEIDGSSLCLQCDMVVHVGGKR--THGRFLL-------------------- 44
BnCBBX83  -----APCCDICENAPAFFYCEIDGSSLCLQCDMVVHVGGKR--THGRFLL-------------------- 44
BnCBBX85  -----APSCDICENAPAFFYCEIDGTSLCLQCDMVVHVGGKR--THRRFLL-------------------- 44
BnABBX6   ----RVRVCEACERAPAAFFCKADAASLCIACDSQIHLVNPLARRHQRVPI-------------------- 47
BnCBBX54  ---ERVRVCEACERAPAAFFCKADAASLCIACDSQVHLANPLARRHQRVPI-------------------- 48
BnABBX42  ----RVRVCQSCERAPAAFFCKADAASLCTACDSQIHSANPLARRHQRVPI-------------------- 47
BnCBBX92  ----RVRVCQSCERAPAAFFCKADAASLCTACDSQIHSANPLARRHQRVPI-------------------- 47
```

```
BnABBX43    ---KRVRVCESCERAPAAFMCEADDVSLCTACDLEVHSANPLARRHQRVPV-------------------- 48
BnCBBX93    ---KRVRVCESCERAPAAFMCEADDVSLCTACDLEVHSANPLARRHQRVPVV------------------- 49
BnABBX49    ---KRVRVCDSCESAPAEFFCKADAASLCTACDAEIHSANPLARRHQRVPI-------------------- 48
BnABBX50    ---KRVRVCDSCESAPAAFFCKADAASLCTACDAEIHSANPLARRHQRVPI-------------------- 48
BnABBX12    -------LCEVCEQAPAAVTCKADAASLCVTCDSDIHSANPLASRHERVPV-------------------- 44
BnCBBX55    -------VCEVCEQAPAAVTCKADAASLCVTCDSDIHSANPLASRHERVPV-------------------- 44
BnCBBX91    -------LCEVCEQAPAAVTCKADAASLCVTCDSDIHSANPLASRHERVPV-------------------- 44
BnABBX15    ---ERVLLCQVCEQSPAHVTCKADAAALCVTCDRDIHSANPLSRRHERVPV-------------------- 48
BnCBBX67    ---ERVWLCQVCEQSPAHVTCKADAAALCVTCDRDIHSANPLSRRHERVPV-------------------- 48
BnABBX23    ---ARVWMCEVCEQAPAHVTCKADAAALCVTCDRDIHSANPLARRHERVPV-------------------- 48
BnCBBX82    ---ARVWMCEVCEQAPAHVTCKADAAALCVTCDRDIHSANPLARRHERVPV-------------------- 48
BnABBX37    ---ARVWMCEVCEQAPAHVTCKADAAALCVTCDRDIHSANPLARRHERVPV-------------------- 48
BnCBBX88    ---ARVWMCEVCEQAPAHVTCKADAAALCVTCDRDIHSANPLARRHERVPV-------------------- 48
BnABBX14    -------VCERCNAQPATVRCVEERVSLCQNCDWSGH---------------------------------- 30
BnCBBX60    -------VCERCNAQPATVRCVEERVSLCQNCDWSG----------------------------------- 29
BnABBX21    -------VCERCNSQPATVRCVEERVSLCQNCDWSGH---------------------------------- 30
BnCBBX71    -------VCERCNAQPATVRCVEERVSLCQNCDWSGH---------------------------------- 30
BnABBX11    -------ICERCNAQPASVRCSDERVSLCQNCDWSGHNNDATSQHKRQSI--------------------- 43
BnCBBX59    -------ICERCNAQPASVRCSDERVSLCQNCDWSGHNNDATSQHKRQSI--------------------- 43
BnABBX24    -------ICERCNAQPASVRCTDERVSLCQNCDWLGH---------------------------------- 30
BnCBBX81    -------ICERCNAQPASVRCTDERVSLCQNCDWLGH---------------------------------- 30
BnABBX17    -------ICDSCGNEPVSVRCFTDDLVLCQECDWDVHGSCS----HVRSAVEGFSGCPSAL---------- 50
BnABBX47    -------ICDSCGNEPVSVRCFTDDLVLCQECDWDVHGSCS----HVRSAVEGFSGCPSAL---------- 50
BnABBX26    -------ICDNCGNEPVSVRCFTDNLVLCQDCDWDVHGSCSVSDAHVRSALEGFSGCPSAL---------- 54
BnCBBX101   -------ICDNCGNEPVSVRCFTDNLVLCQDCDWDVHGSCSVSDAHVRSAVEGFSGCPSAL---------- 54
BnABBX34    -------ICDNCGSEPVSVRCFTDSLVLCQECDWDVHGSCSVSDAHVRSAIEGFTGCPSAL---------- 54
BnCBBX62    -------ICDNCGSEPVSVRCFTDSLVLCQECDWDVHGSCSVSDAHVRSVVEGFTGCPSAL---------- 54
BnABBX18    -------LCDSCDDSPSSVFCDTESSVLCQNCDWQHHTSAS--SLHSRRPL-------------------- 42
BnCBBX65    -------LCDSCDDSPSSVFCDTESSVLCQNCDWQ------------------------------------ 28
```
