# Supplementary material for: Genome-Wide Identification, Evolution, and Comparative Analysis of B-Box Genes in Brassica rapa, B. oleracea, and B. napus and Their Expression Profiling in B. rapa in Response to Multiple Hormones and Abiotic Stresses
Source: Int J Mol Sci. 2021 Sep 26;22(19):10367. doi: 10.3390/ijms221910367 (PMC8509055; doi:10.3390/ijms221910367)
Supplement: Supplementary file 1 [file ijms-22-10367-s001.zip › Suppl Fig. 1c_.pdf]

```
                       10        20        30        40        50        60        70
            ....|....|....|....|....|....|....|....|....|....|....|....|....|...
BrBBX8      ------------REARVMRYREKKKTR--KFEKRVRYASRKAYAETRPRIKGRFVKRE--------------- 44
BrBBX49     ------------RKARVMRYREKKKTR--KFEKTIRYASRKEYAEKRPRIKGRFAKRN--------------- 44
BrBBX7      --------------KRVRVCDSCESAPA-AFFCKADAASLCTACDAEIHSANPLARRHQRVPI---------- 48
BrBBX50     DAQVHSANRVASRHKRVRVCESCERAPA-AFMCEADDVSLCTACDLEVHSANPLARRHQRVPV---------- 62
BrBBX26     ------------REARVMRYREKRKNR--KFEKTIRYASRKAYAEMRPRIKGRFAKR---------------- 43
BrBBX42     ------------REARVMRYREKRKNR--KFEKTIRYASRKAYAEMRPRIKGRFAKR---------------- 43
BrBBX19     ------------REARVLRYREKRKNR--KFEKTIRYASRKAYAEVRPRIKGRFAKR---------------- 43
BrBBX10     ------------REARVLRYREKRKNR--KFEKTIRYASRKAYAESRPRIKGRFAKR---------------- 43
BrBBX29     ------------RDNAMQRYKEKRKTR--RYDKTIRYESRKATADTRLRVKGRFVKA---------------- 43
BrBBX51     ------------RGNAMQRYKEKRKNR--RYDKTIRYESRKARADTRLRVKGRFVKA---------------- 43
BrBBX39     ------------RDNAMQRYKEKRKTR--RYDKTIRYESRKARADTRLRVKGRFVKA---------------- 43
BrBBX21     ------------RNSALSRYKAKKKSR--RYEKHIRYESRKVRAETRTRIRGRFAKS---------------- 43
BrBBX6      ------------RNNAVMRYKEKKKAR--KFDKTVRYASRKARADVRRRVKGRFIKAG--------------- 44
BrBBX25     ------------RNNAVMRYKEKKKAR--KFDKTVRYASRKARADVRRRVKGRFVKAG--------------- 44
BrBBX17     ------------RNNAVMRYKEKKKAR--KFDKRVRYASRKARADVRRRVKGRFVKAG--------------- 44
BrBBX15     --------HATTRNNAVLRYKEKKKAR--KFDKRVRYVSRKERADVRRRVKGR-------------------- 43
BrBBX27     ------------RNNAVIRYKEKKKAR--KFDKRVRYVSRKERADVRRRVKGRFVKSG--------------- 44
BrBBX5      ------------RNEAKLRYKEKKLKR--TFGKQIRYASRKARADTRKRVKGRFVKAG--------------- 44
BrBBX48     ----------------------------------IRYESRKSSADTKKRLKGRFTKAD--------------- 24
BrBBX12     ------------REARVSRYREKRRTR--LFSKKIRYEVRKLNAEKRPRMKGRFVKRS--------------- 44
BrBBX33     ------------REARVSRYREKRRTR--LFSKKIRYEVRKLNAEKRPRMKG--------------------- 38
BrBBX30     ------------REARVSRYREKRRTR--LFSKKIRYEVRKLNAEKRPRMKGRFVKRA--------------- 44
BrBBX43     ------------REARVSRYREKRRTR--LFSKKIRYEVRKLNAEKRPRMKGRFVKR---------------- 43
BrBBX40     ------------REASVSRYREKKKTR--SFTKTIRYE----------------------------------- 24
BrBBX23     ------------REARVWRYRDKRKNR--LFEKKIRYEVRKVNADKRPRIKGRFVRRS--------------- 44
BoBBX7      ------------REARVMRYREKKKTR--KFEKRVRYASRKAYAETRPRIKGRFVKRE--------------- 44
BoBBX50     ------------------------------------YASRKEYAEKRPRIKGRFAKRN--------------- 22
BoBBX6      ------------REARVLRYREKKKRR--KFEKTIRYASRKAYAEVRPRIKGRFAKR---------------- 43
BoBBX51     ------------REARVLRYREKKKRR--KFEKTIRYASRKAYAERRPRINGRFAKMS--------------- 44
BoBBX8      ------------REARVLRYREKRKNR--KFEKTIRYASRKAYAESRPRIKGRFAKR---------------- 43
BoBBX49     ------------REARVLRYREKRKNR--KFEKTIRYASRKAYAESRPRIKGRFAKR---------------- 43
BoBBX38     --------------------REKRKNR--KFEKTIRYASRKAYAEMRPRIKGRFAKR---------------- 35
BoBBX46     ------------REARVMRYREKRKNR--KFEKTIRYASRKAYAEMRPRIKGRFAKR---------------- 43
BoBBX22     ------------REARVLRYREKRKNR--KFEKTIRYASRKAYAEVRPRIKGRFAKR---------------- 43
BoBBX10     ------------REARVSRYREKRRTR--LFSKKIRYEVRKLNAEKRPRMKGRFVKRS--------------- 44
BoBBX30     ------------REARVSRYREKRRTR--LFSKKIRYEVRKLNAEKRPRMKGRFVKRS--------------- 44
BoBBX35     ------------REARVSRYREKRRTR--LFSKKIRYEVRKLNAEKRPRMKGRFVKRA--------------- 44
BoBBX36     ------------REARVSRYREKRRTR--LFSKKIRYEVRKLNAEKRPRMKGRFVKRA--------------- 44
BoBBX23     ------------REARVSRYREKRRTR--LFSKKIRYEVRKLNAEKRPRMKGRFVKR---------------- 43
BoBBX16     ------------REARVSRYREKKKTR--SFTKTIRYEVRKLNAENKPRMKGRFVKRA--------------- 44
BoBBX24     ------------REARVWRYRDKRKNR--LFEKEIRYEVRKVNADKRPRMKGRFVRRS--------------- 44
BoBBX4      ------------RNEAKLRYKEKKLKR--TFGKQIRYASRKARADTRKRVKGRFVKAG--------------- 44
BoBBX45     -----------------------------KFGKKIRYESRKSSADTKKRLKGRFTKADAEYDYDPRANNTTKE 44
BoBBX5      --------------------------------------SRKARADVRRRVKGRFIKAGEAYD----------- 24
BoBBX27     -------SHSVTRNNAVMRYKEKKKAR--KFDKTVRYASRKARADVRRRVKGR-------------------- 44
BoBBX14     ------------RNNAVMRYKEKKKAR--KFDKRVRYASRKARADVRRRVKGRFVKAG--------------- 44
BoBBX13     ------------RNNAVLRYKEKKKAR--KFDKRVRYVSRKERADVRRRVKGRFVKSG--------------- 44
BoBBX37     ------------RNNAVIRYKEKKKAR--KFDKRVRYVSRKERADVRRRVKGRFVKSG--------------- 44
BoBBX17     ------------RDNAMQRYKEKRKNR--RYDKTIRYESRKARADTRLRVKGRFVKA---------------- 43
BoBBX34     ------------RDNAMQRYKEKRKTR--RYDKTIRYESRKATADTRLRVKGRFVKA---------------- 43
BoBBX52     ------------RGNAMQRYKEKRKNR--RYDKTIRYESRKARADTRLRVKGRFVKA---------------- 43
BoBBX20     ------------RNSALSRYKAKKKSR--RYEKHIRYESRKVRAESRTRIRGRFAKS---------------- 43
BnABBX8     ------------REARVSRYREKRRTR--LFSKKIRYEVRKLNAEKRPRMKGRFVKRS--------------- 44
BnCBBX56    ---------RLDYESVISTWGGQGTPWTAREPPQIDLNMLCCPTDSMVESGG--------------------- 43
BnABBX31    ------------REARVSRYREKRRTR--LFSKKIRYEVRKLNAEKRPRMKGRFVKRA--------------- 44
BnCBBX97    ------------REARVSRYREKRRTR--LFSKKIRYEVRKLNAEKRPRMKGRFVKRA--------------- 44
BnCBBX80    ------------REARVSRYREKRRTR--LFSKKIRYEVRKLNAEKRPRMKGRFVKRA--------------- 44
BnABBX46    ------------REARVSRYREKRRTR--LFSKKIRYEVRKLNAEKRPRMKGRFVKR---------------- 43
BnCBBX72    ------------REARVSRYREKRRTR--LFSKKIRYEVRKLNAEKRPRMKGRFVKR---------------- 43
BnABBX35    ------------REASVSRYREKKKTR--SFTKTIRYE----------------------------------- 24
BnCBBX94    ------------REARVSRYREKKKTR--SFTKTIRYE----------------------------------- 24
BnABBX22    ------------REARVWRYRDKRKNR--LFEKKIRYE----------------------------------- 24
BnABBX6     ------------REARVMRYREKKKTR--KFEKRVRYASRKAYAETRPRIKGRFVKRE--------------- 44
BnCBBX54    ------------REARVMRYREKKKTR--KFEKRVRYASRKAYAETRPRIKGRFVKRE--------------- 44
BnABBX42    -------------KARVMRYREKKKTR--KFEKTIRYASRKEYAEKRPRIKGRFAKRN--------------- 43
BnCBBX92    ----------------VMRYREKKKTR--KFEKRIRYASRKEYAEKRPRIKGRFAKRN--------------- 40
BnABBX43    ------------REARVLRYREKKKRR--KFEKTIRYASRKAYAERRPRINGRFAKISE-------------- 45
BnCBBX93    ------------REARVLRYREKKKRR--KFEKTIRYASRKAYAERRPRINGRFAKMS--------------- 44
BnABBX49    ------------REARVLRYREKKKRR--KFEKTIRYASRKAYAEVRPRIKGRFAKR---------------- 43
BnABBX50    ------------REARVLRYREKKKRR--KFEKTIRYASRKAYAEVRPRIKGRFAKR---------------- 43
```

```
BnABBX12    ------------REARVLRYREKRKNR--KFEKTIRYASRKAYAESRPRIKGRFAKR---------------- 43
BnCBBX55    ------------REARVLRYREKRKNR--KFEKTIRYASRKAYAESRPRIKGRFAKR---------------- 43
BnCBBX91    ------------REARVLRYREKRKNR--KFEKTIRYASRKAYAESRPRIKGRFAKR---------------- 43
BnABBX15    ------------REARVLRYREKRKNR--KFEKTIRYASRKAYAEVRPRIKGRFAKR---------------- 43
BnCBBX67    ------------REARVLRYREKRKNR--KFEKTIRYASRKAYAEVRPRIKGRFAKR---------------- 43
BnABBX23    ------------REARVMRYREKRKNR--KFEKTIRYASRKAYAEMRPRIKGRFAKR---------------- 43
BnCBBX82    ------------REARVMRYREKRKNR--KFEKTIRYASRKAYAEMRPRIKGRFAKR---------------- 43
BnABBX37    ------------REARVMRYREKRKNR--KFEKTIRYASRKAYAEMRPRIKGRFAKR---------------- 43
BnCBBX88    ------------REARVMRYREKRKNR--KFEKTIRYASRKAYAEMRPRIKGRFAKR---------------- 43
BnABBX45    ----------------------------------IRYESRKSSADTKKRLKGRFTKAD--------------- 24
BnCBBX86    ----------------------------------IRYESRKSSADTKKRLKGRFTKAD--------------- 24
BnABBX4     ------------RNEAKLRYKEKKLKR--TFGKQIRYASRKARADTRKRVKGRFVKAG--------------- 44
BnCBBX100   ------------RNEAKLRYKEKKLKR--TFGKQIRYASRKARADTRKRVKGRFVKAG--------------- 44
BnABBX14    ------------RNNAVMRYKEKKKAR--KFDKRVRYASRKARADVRRRVKGRFVKAG--------------- 44
BnCBBX60    ------------RNNAVMRYKEKKKAR--KFDKRVRYASRKARADVRRRVKGRFVKAG--------------- 44
BnABBX21    ------------RNNAVMRYKEKKKAR--KFDKTVRYASRKARADVRRRVKGRFVKAG--------------- 44
BnCBBX71    ------------RNNAVMRYKEKKKAR--KFDKTVRYASRKARADVRRRVKG--------------------- 38
BnCBBX52    ------------RNNAVMRYKEKKKAR--KFDKTVRYASRKARADVRRRVKGRFIKAG--------------- 44
BnABBX11    ------------RNNAVLRYKEKKKAR--KFDKRVRYVSRKERADVRRRVKGRFVKSG--------------- 44
BnCBBX59    ------------RNNAVLRYKEKKKAR--KFDKRVRYVSRKERADVRRRVKGRFVKSG--------------- 44
BnABBX24    ------------RNNAVIRYKEKKKAR--KFDKRVRYVSRKERADVRRRVKGRFVKSG--------------- 44
BnCBBX81    ------------RNNAVIRYKEKKKAR--KFDKRVRYVSRKERADVRRRVKGRFVKSG--------------- 44
BnABBX17    ------------RGNAMQRYKEKRKNR--RYDKTIRYESRKARADTRLRVKGRFVKA---------------- 43
BnABBX47    ------------RGNAMQRYKEKRKNR--RYDKTIRYESRKARADTRLRVKGRFVKA---------------- 43
BnABBX26    ------------RDNAMQRYKEKRKTRS-RYDKTIRYESRKATADTRLRVKGRFVKA---------------- 44
BnCBBX101   ------------RDNAMQRYKEKRKTRS-RYDKTIRYESRKATADTRLRVKGRFVKA---------------- 44
BnABBX34    -----------NRDNAMQRYKEKRKNR--RYDKTIRYESRKARADTRLRVKGRFVKAT--------------- 45
BnABBX18    ------------RNSALSRYKAKKKSR--RYEKHIRYESRKVRAESRTRIRGRFAKS---------------- 43
```
