# Supplementary figures and images for: Genome-Wide Identification, Evolution, and Comparative Analysis of B-Box Genes in Brassica rapa, B. oleracea, and B. napus and Their Expression Profiling in B. rapa in Response to Multiple Hormones and Abiotic Stresses
Source: Int J Mol Sci. 2021 Sep 26;22(19):10367. doi: 10.3390/ijms221910367 (PMC8509055; doi:10.3390/ijms221910367)

a

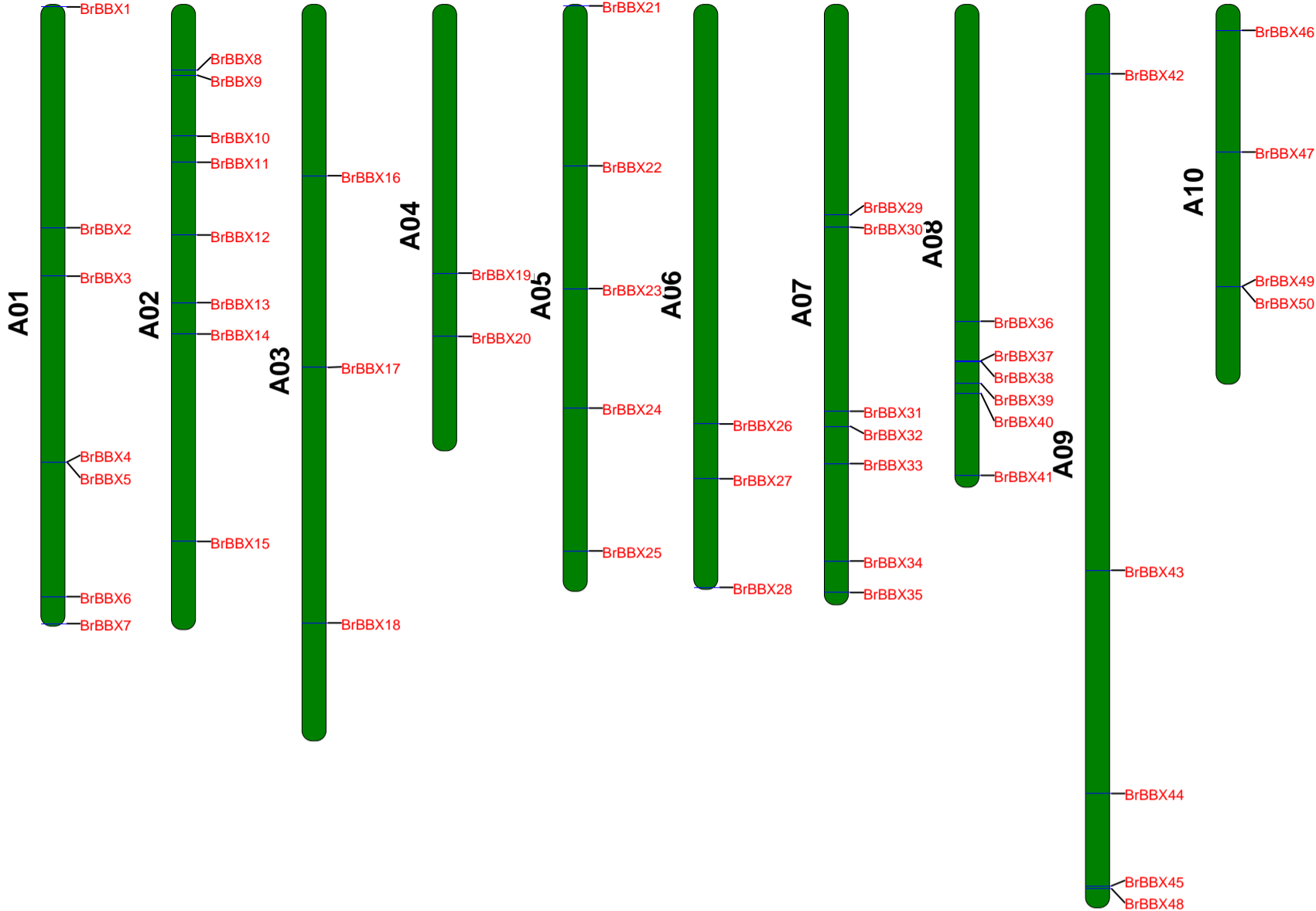

b

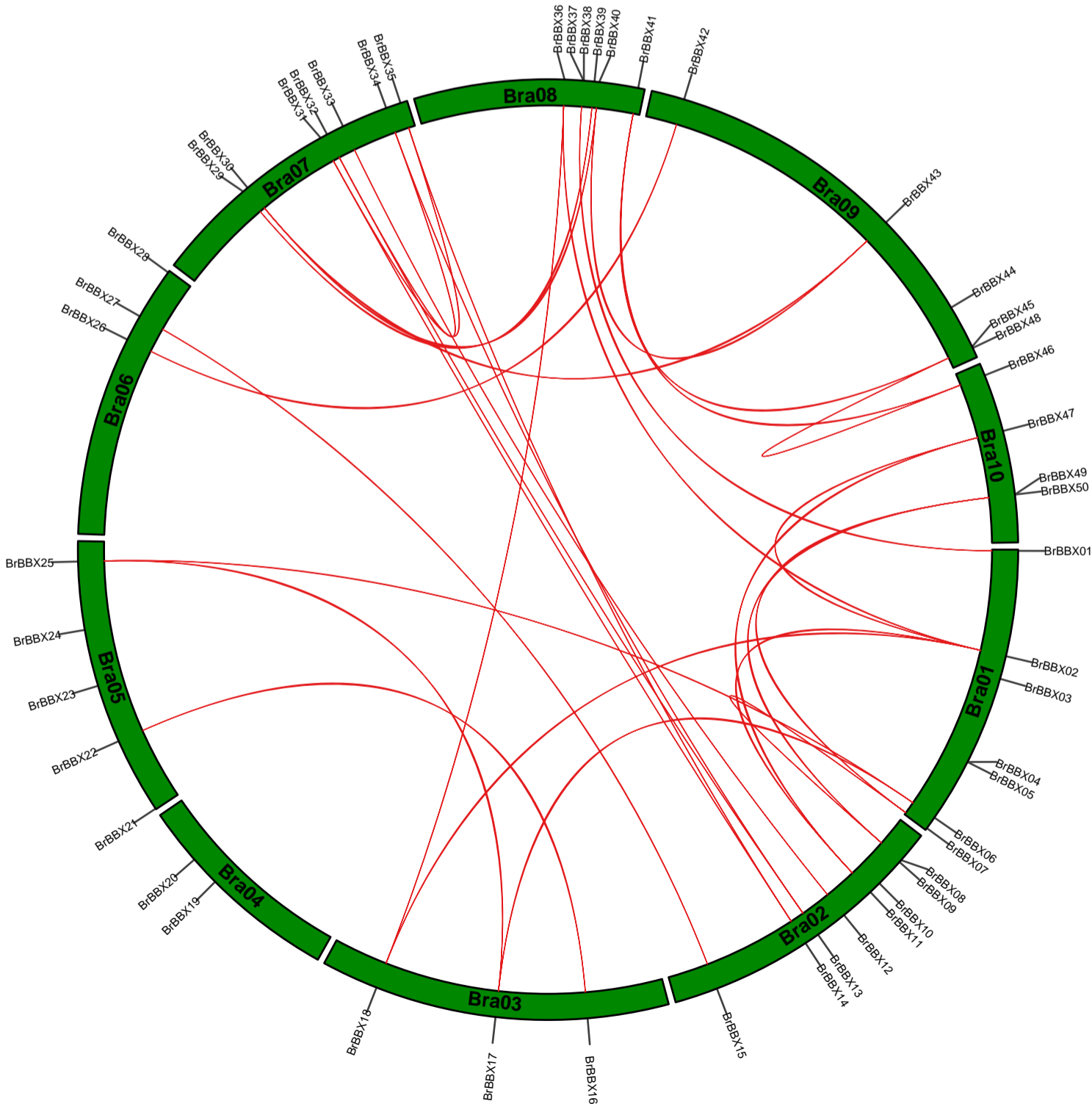

Supplement: Supplementary file 1 [file ijms-22-10367-s001.zip › Suppl Fig. 2.pdf]

a

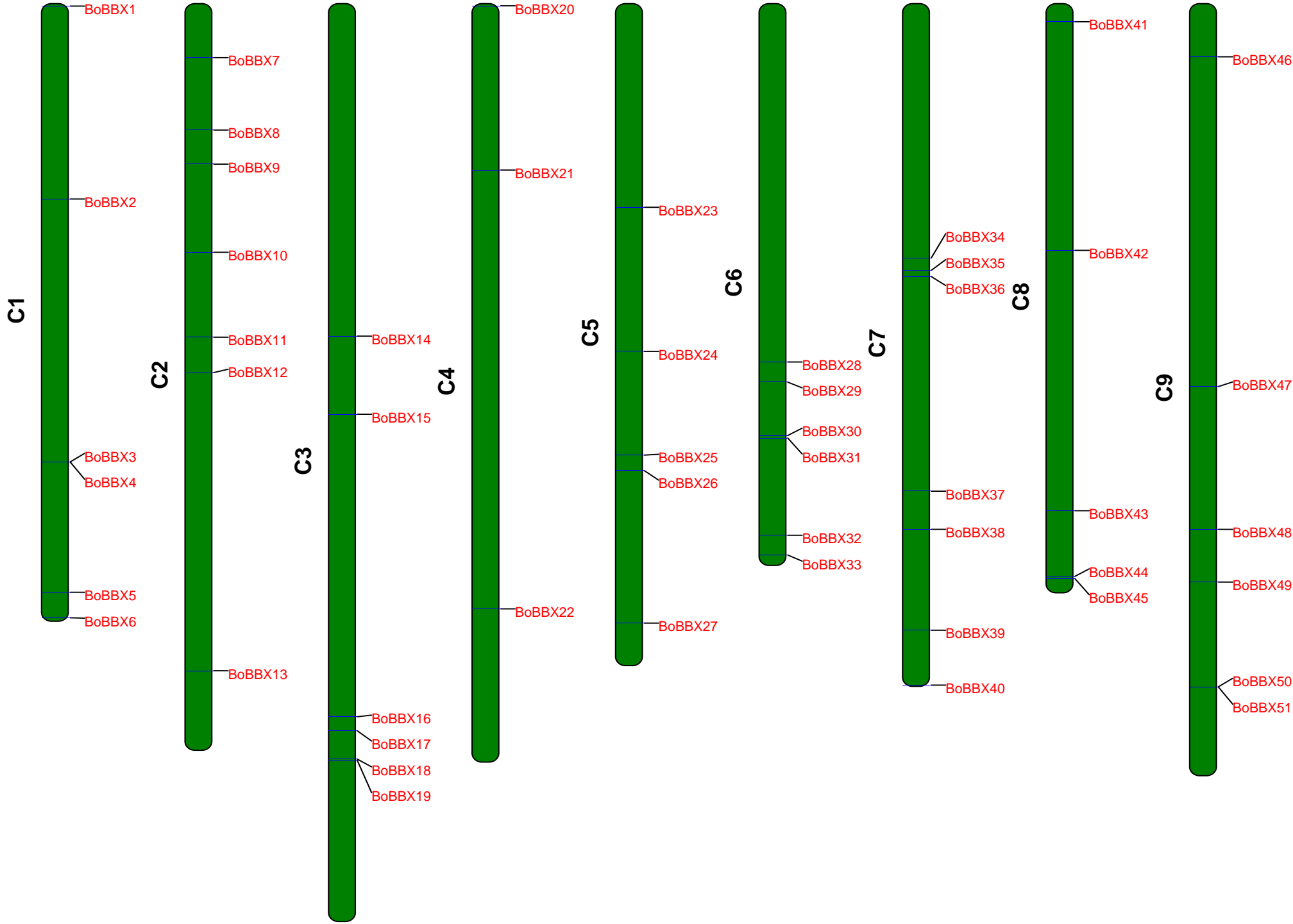

b

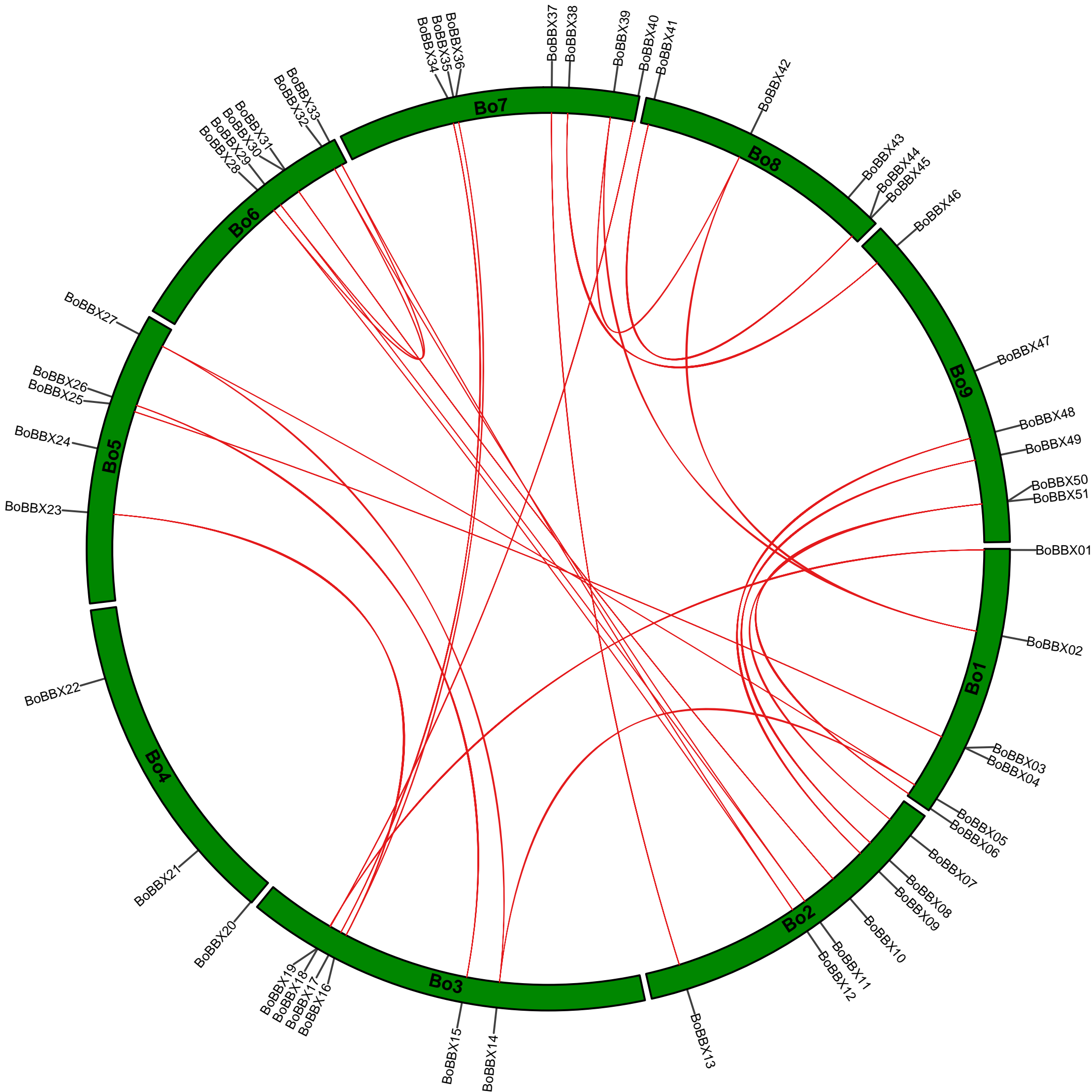

Supplement: Supplementary file 1 [file ijms-22-10367-s001.zip › Suppl Fig. 3.pdf]

a

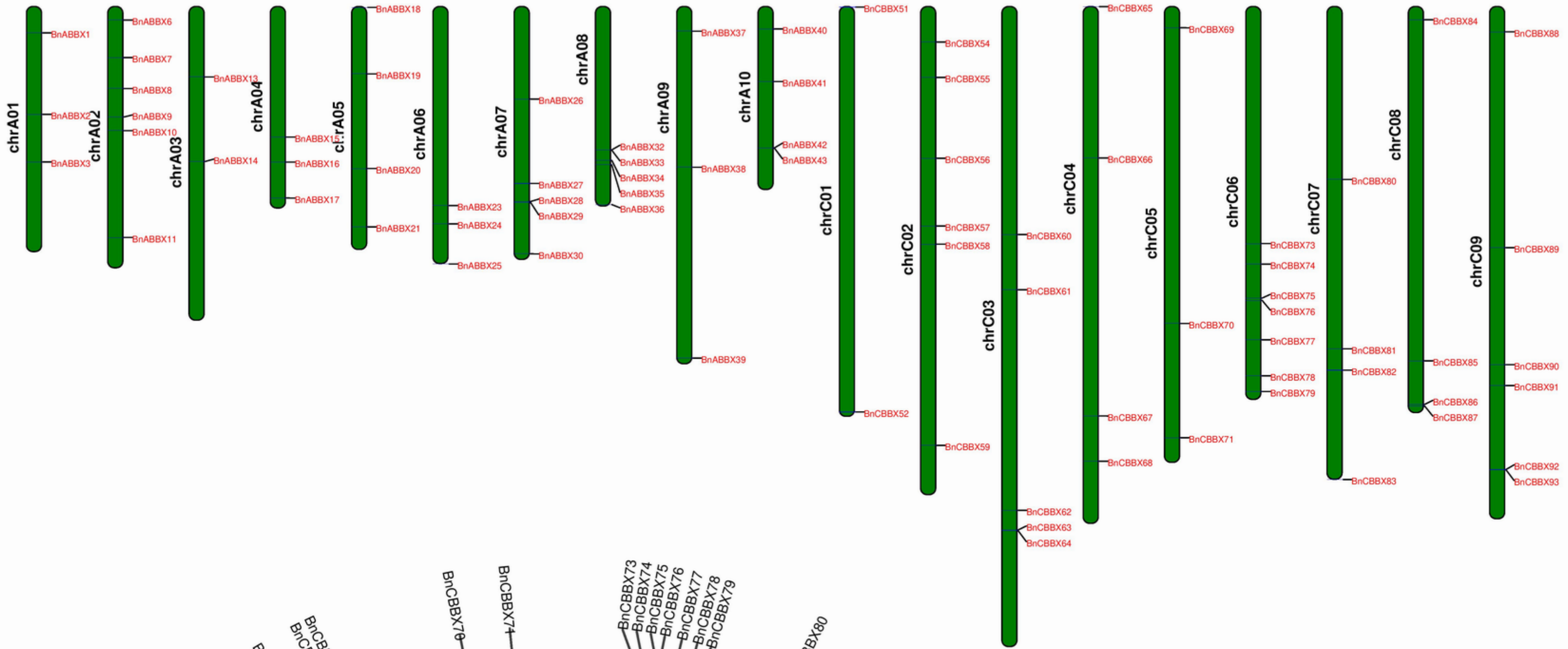

b

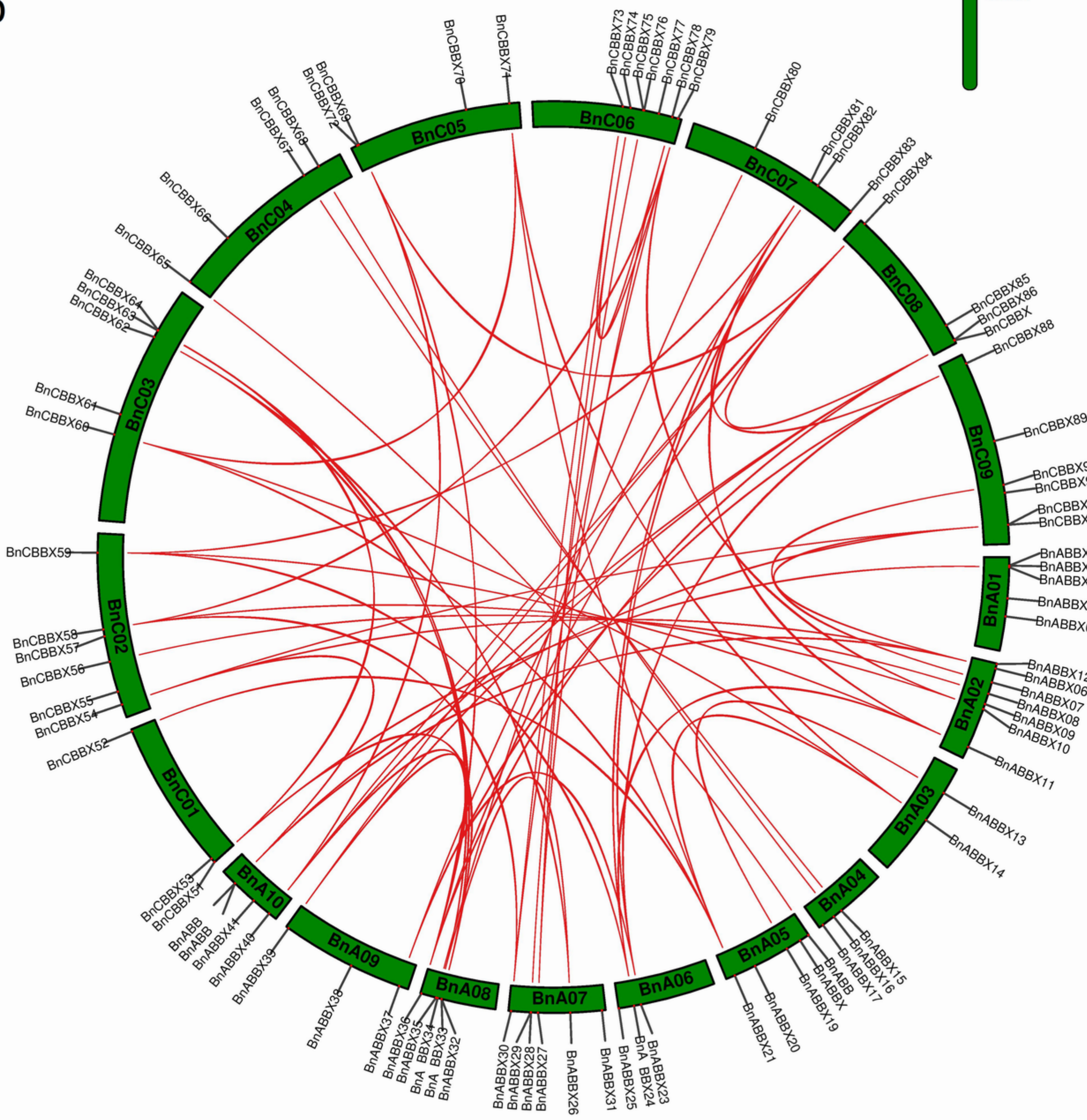

Supplement: Supplementary file 1 [file ijms-22-10367-s001.zip › Suppl Fig. 4.pdf]
